# Theoretical prediction of broadband ambient light optogenetic vision restoration with ChRmine and its mutants

**DOI:** 10.1038/s41598-024-62558-2

**Published:** 2024-05-21

**Authors:** Himanshu Bansal, Gur Pyari, Sukhdev Roy

**Affiliations:** Department of Physics and Computer Science, Dayalbagh Educational Institute, Agra, 282005 India

**Keywords:** Optogenetics, Vision restoration, Channelrhodopsin, ChRmine, bReaChES, CoChR, PsCatCh2.0, Broadband activation, Sunlight optogenetics, Biological models, Optogenetics, Optics and photonics, Eye diseases, Macular degeneration

## Abstract

Vision restoration is one of the most promising applications of optogenetics. However, it is limited due to the poor-sensitivity, slow-kinetics and narrow band absorption spectra of opsins. Here, a detailed theoretical study of retinal ganglion neurons (RGNs) expressed with ChRmine, ReaChR, CoChR, CatCh and their mutants, with near monochromatic LEDs, and broadband sunlight, halogen lamp, RGB LED light, and pure white light sources has been presented. All the opsins exhibit improved light sensitivity and larger photocurrent on illuminating with broadband light sources compared to narrow band LEDs. ChRmine allows firing at ambient sunlight (1.5 nW/mm^2^) and pure white light (1.2 nW/mm^2^), which is lowest among the opsins considered. The broadband activation spectrum of ChRmine and its mutants is also useful to restore color sensitivity. Although ChRmine exhibits slower turn-off kinetics with broadband light, high-fidelity spikes can be evoked upto 50 Hz. This limit extends upto 80 Hz with the improved hsChRmine mutant although it requires double the irradiance compared to ChRmine. The present study shows that ChRmine and its mutants allow activation of RGNs with ambient light which is useful for goggle-free white light optogenetic retinal prostheses with improved quality of restored vision.

## Introduction

Blindness caused from retinal degeneration affects millions of people worldwide^1^. In patients affected with retinal degenerative diseases that include retinitis pigmentosa and macular degeneration, the photoreceptor cell death breaks the conversion process of light signal to electrical signal in the retina, and thus, it leads to complete loss of vision. However, the remaining retinal circuitry retains functionality and connections to the brain via the optic nerve such that direct electrical stimulation of retinal neurons restores visual sensation^2^. At present, advanced electrical prosthetic devices are used to restore vision in humans. However, the method has fundamental limitations that include invasiveness and poor spatial resolution^3,4^.

Optogenetics has revolutionized neuroscience by allowing minimally invasive circuit-specific activation, suppression, and bidirectional control of neuronal activity at unprecedented spatiotemporal resolution^4,5^. Restoring visual function in the degenerated retina by direct optogenetic excitation of neurons in the inner layers of the retina is an emerging technology^6–8^. Optogenetic stimulation in the retina does not only overcome the challenges associated with electrical stimulation but also provides quality vision^9,10^. Using optogenetics, light response in blind mice was first restored by expressing channelrhodopsin-2 (ChR2) in thalamic projecting neurons^11^. However, it is reported that high-intensity blue light, required to evoke spiking in ChR2-expressing retinal ganglion neurons (RGNs) causes photochemical damage and phototoxicity in human retina and retinal pigment epithelium^12,13^.

A new blue light-sensitive ChR variant, named CoChR has been discovered, which shows improved expression, large photocurrent and relatively fast off kinetics than ChR2^14^. Recently, two new engineered variants of CoChR, namely CoChR-LC and CoChR-3M, with improved light-sensitivity have been reported^15^. These mutants still exhibit slower kinetics, although their off-kinetics has been optimized through site-directed mutagenesis. To reduce the irradiance requirement, Ca^2+^ permeable opsin, named CatCh, has been used in RGNs^16^. Although the irradiance to activate CatCh-expressing neurons is below safety threshold, it is still high in comparison to natural daylight^16^. Recently, PsCatCh2.0, an engineered mutant of CatCh, has shown inherently high Ca^2+^ and Na^+^ conductance, and provided excitation of RGNs at ~ 10^12 ^ photons. mm^−2^ s^−1^. However, it has a firing limit upto 32 Hz^17^.

The retina has higher irradiance safety thresholds for red light^18,19^. Therefore, intense efforts have been made to discover new opsins exhibiting red-shifted activation spectrum. A red-activable mutant of ChR, named ReaChR, having its peak activation at 590 nm, has been used to restore sensitivity in RGNs in blind rd1 mice, primate retina, and post-mortem human retinae^20^. However, ReaChR exhibits very slow kinetics, enabling firing only upto 30 Hz. Further, red-shifted opsins with larger photocurrent, fast kinetics, and higher sensitivity are constantly being discovered and engineered for enhancing safety and feasibility^20^. To generate high-frequency spiking, a fast red-shifted opsin, named ChrimsonR has also been reported that was first shown to restore vision in non-human primate in a pulsed laser-induced retinal generation model^21^. Vision restoration using optogenetics has been first shown in nonhuman primate expressing ChrimsonR in RGNs in pulsed laser-induced retinal degeneration model^13^. Thereafter, a clinical study in a human subject successfully demonstrated partial recovery of vision using ChrimsonR, demonstrating the effectiveness and safety of optogenetics^22^.

In optogenetics, natural light-sensitive proteins play a central role^5,23^. For optogenetic excitation of retinal neurons, unnatural bright light intensities are required due to poor light-sensitivity and narrow band absorption spectra of opsins. Although experiments have shown that the light levels required with new opsins are safe, the activation threshold is still high in comparison to daylight intensity^19,21,24^. Therefore, the use of extraocular devices such as optoelectronic goggles become necessary to convert the natural scene into high-intensity monochromatic light pulses for sufficient excitation of opsin-expressing retinal neurons^22^. These extraocular devices are also needed to compensate for the loss of visual processing of bypass part of the visual processing pathways^6^. However, such devices encounter significant difficulties in eye tracking and registering micromovements of the devices relative to the face^6^. Ideally, the targeted retinal neurons should respond to the natural daylight intensity, which is of the order of ~ µW/mm^2 ^^25^. To overcome these challenges, intense research efforts are going on to discover and engineer new opsins for enhanced light-sensitivity and broadband activation spectra^26,27^. A fast red-shifted variant of ReaChR, named bReaChES was also generated^28^. Recently, bReaChES has enabled spiking at light levels consistent with bright indoor lighting, alongwith sufficient firing limit up to 50 Hz on illuminating with red light LED having bandwidth ~ 24 nm^28^. Most recently, a new mutant, namely ex3mV1Co from the modified Volvox channelrhodopsin-1 (mVChR1), has been reported that has enabled excitation of retinal neurons at very low narrow band light intensities ~ µW/mm^2 ^^25^.

The illumination strategy in optogenetics is primarily restricted to narrow-band light sources^4^. The recent approaches to restoring vision are focused on using arrays of intense narrow-band light sources to activate opsin-sensitized cells in the retina^22^. However, the resolution of restored vision is limited by the density of light sources in the array. Ideally, the treated retina should respond to broadband white light at daylight levels. A few studies have reported that broadband activation of opsins results in significantly larger photocurrent and higher sensitivity than narrow band light sources^26^. Further, an opsin construct having broadband activation spectra was also designed by fusion of three opsins having their absorption peak in blue, green, and red regions. This opsin is also called *white opsin*^27,29,30^. The study has shown that broadband activation of the white opsin results in significantly improved photocurrent at lower light-intensities^30^. However, the delivery of a large fusion construct is challenging and can lead to immune reactions^6,25^. Therefore, opsins having broadband absorption spectra, higher sensitivity, and large photocurrent are desired.

Recently, new opsins with improved sensitivity, photocurrent amplitude, and broader activation spectrum have been reported. ChRmine, a bacteriorhodopsin-like cation ChR derived from *Rhodomonas lens*, also attributed to the marine ciliate *Tiarina fusus,* is one of the most promising newly discovered opsin^31,32^. The large photocurrent (~ 4 nA) and higher sensitivity of ChRmine results in the excitation of neurons at ultra-low light intensities^32,33^. A recent study on cardiac optogenetics has shown that ChRmine enables contractions of mouse heart at very low light intensities ~ 0.1 mW/mm^2 ^^34^. More recently, new variants of ChRmine, namely hsChRmine, rsChRmine, and frChRmine have been reported^35^. hsChRmine exhibits accelerated-kinetics, whereas rsChRmine and frChRmine have enhanced red-light sensitivity. These mutants retain high photosensitivity, broadband activation spectrum, and fast kinetics, similar to ChRmine^35,36^. Although, there are a few opsins that include bReaChES, ChRmine, and its mutants, which have shown significantly improved light sensitivity and sufficiently fast kinetics under narrow band light sources, their response to broadband light sources has not been studied as yet.

Computational optogenetics has greatly contributed to our understanding of complex dynamics of single or multiple opsin-expressing neurons in response to optical stimulations^37,38^. Furthermore, it has helped in reducing time, effort and cost by providing an optimized set of photostimulation and physiological conditions with exploration of light to spike process under continuous and pulsed illumination^38–43^. Recently, computational modeling of optogenetic visual cortical prosthetic systems has been reported, which provides better understanding of the impact of optogenetic stimulation on cortical dynamics and encoding of visual stimuli in the cortex^44^. More recently, a computational model of monochromatic optogenetic excitation of ChRmine in RGNs has been reported by our group^45^. A detailed comparative study of the performance of different opsins is required to find the best opsin for optogenetic vision restoration, and to guide the design of new opsins.

The aim of this paper is to theoretically investigate, (i) the photoresponse of RGNs expressed with different opsins, namely, ChRmine, rsChRmine, hsChRmine, frChRmine, ReaChR, bReaChES, CoChR, CoChR-LC, CoChR-3M, CatCh, and PsCatch2.0 on illuminating with broadband (sunlight, halogen lamp light, RGB LED based white light, and pure white light) light sources and compare their response with narrow band light sources (light-emitting diodes (LEDs)), and (ii) the combined effect of high-sensitivity, large photocurrent, fast-kinetics and broadband activation spectra of new opsins to achieve excitation at ultra-low light intensity as well as sufficiently high-frequency for high-quality, goggle-free optogenetic vision restoration.

## Results

**Figure 1:**
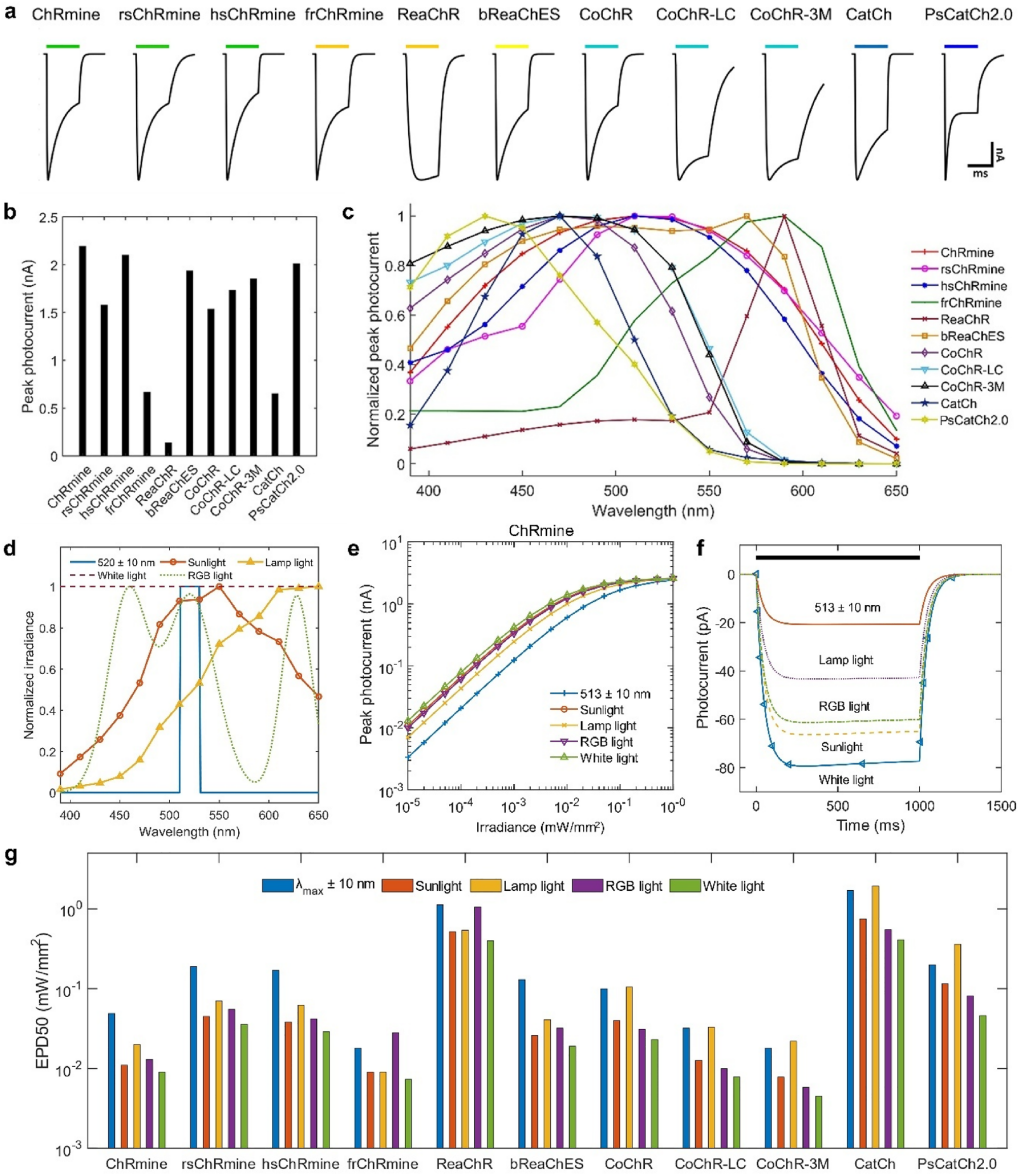
Theoretical simulation of photocurrent and light-sensitivity in different opsins on illuminating with near monochromatic and broadband light sources. (**a**) Variation of normalized photocurrent with time in different opsins on illuminating with 1 s LED light pulse at the respective peak activation wavelengths for each opsin, having 20 nm bandwidth at 1 × 10^15^ photons mm^−2^ s^−1^, and (**b**) correspoding peak photocurrent amplitude. (**c**) Variation of normalized peak photocurrent with wavelength under similar conditions as in (**a**). (**d**) Normalized spectral irradiance of different light sources. (**e**) Variation of peak photocurrent with irradiance in ChRmine for different light sources at 1 s light pulse. (**f**) Variation of photocurrent with time in ChRmine on illuminating with different light sources at 0.1 µW/mm^2^. (**g**) Effective power density to achieve 50% photocurrent (EPD50) for different opsins on activating with different light sources.

The simulated photocurrents in different opsins on illuminating with LEDs at their peak activation wavelengths having 20 nm bandwidth are shown in Fig. 1a. At same photon flux density and light pulse duration, each opsin exhibits unique photocurrent kinetics. The photocurrent amplitude of these opsins is compared in Fig. 1b. ChRmine results in the largest photocurrent among the ChRs, whereas the photocurrents in hsChRmine, bReaChES, CoChR-3M, and PsCatCh2.0 are also very large in comparison to other opsins. The spectral sensitivity of photocurrent in each opsin over a broad range of wavelength, 390–650 nm, is shown in Fig. 1c. The photocurrents in ChRmine, rsChRmine, hsChRmine, and bReaChES are effectively large over a broad spectral range. Therefore, broadband light can provide excitation of these opsins at lower irradiances.

For broadband light sources, four types of light sources, namely sunlight, lamp light, RGB light and pure white light have been considered. Figure 1d shows the normalized spectral intensity of these broadband light sources and an LED having 20 nm bandwidth (λ = 520 ± 10 nm). The maximum of the sunlight spectrum at ~ 550 nm is very close to the absorption maxima of ChRmine, rsChRmine, hsChRmine, and bReaChES (Fig. 1c,d). The lamp light used here has red-shifted spectral maxima at ~ 600 nm, which lies in the maximum sensitivity range for frChRmine, and ReaChR. The RGB light source has three distinct peaks at 458 nm, 521 nm, and 628 nm in blue, green and red spectral regions, respectively^46^. A pure white light having flat intensity profile over the whole visible spectral range has also been included to study the response under ideal conditions (Fig. 1d).

On illuminating with the LED and different broadband light sources, the variation of peak photocurrent in ChRmine with irradiance is shown in Fig. 1e. Similar variation for other opsins is shown in Supplementary Fig. S1. As it is evident, the photocurrent in ChRmine and other opsins saturates at lower irradiances for broadband light sources than for LED (Fig. 1e, Supplementary Fig. S1). Also, the difference in the photocurrent amplitude with different sources is larger at lower irradiances (Fig. 1e). The photocurrent in ChRmine on illuminating with each of the light sources at 0.1 µW/mm^2^ is shown in Fig. 1f. The amplitude of photocurrent for pure white light (79.4 pA) is four times higher than for LED (20.8 pA) at the same irradiance (0.1 µW/mm^2^) (Fig. 1f).

The effective power density to achieve 50% photocurrent (EPD50) for each opsin and light source is shown in Fig. 1g. In all the opsins, EPD50 is the lowest for pure white light, indicating improved photosensitivity of opsins at broadband light sources (Fig. 1g). For other types of broadband sources, EPD50 attains different values depending on overlapping of their activation spectrum with the light source. The red-shifted opsins that include ChRmine, hsChRmine, and rsChRmine exhibit lower EPD50 for sunlight source, whereas the blue-shifted opsins that include CoChR, CoChR-LC, CoChR-3M, CatCh, and PsCatCh2.0 have lower EPD50 for RGB light. The peak photocurrent in different opsins at EPD50 for different light sources is compared in Supplementary Fig. S2. As it is evident, all opsins have the largest photocurrent for pure white light source (Supplementary Fig. S2).

**Figure 2:**
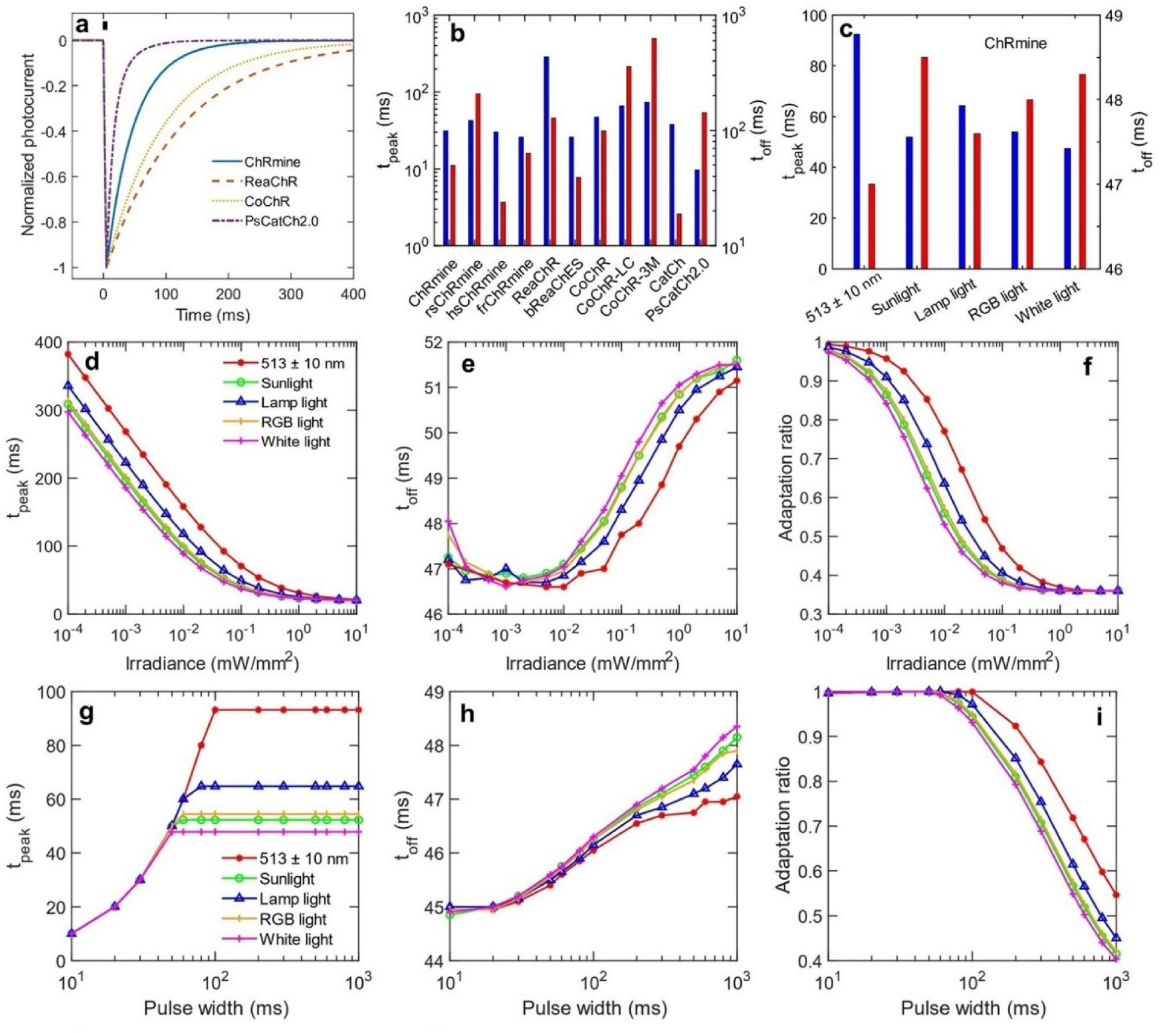
Theoretical simulation of photourrent kinetics in different opsins on illuminating with near monochromatic and broadband light sources. (**a**) Normalized photocurrent in different opsins on activating with a 5 ms LED pulse at 1 mW/mm^2^, and (**b**) corresponding t_off_, time to reduce the photocurrent to 1/e after light-off and t_peak_, time to achieve peak photocurrent after light-on for 1 s light pulse. (**c**) t_peak_ and t_off_ in ChRmine on illuminating with different light sources at 0.05 mW/mm^2^ for 1 s light pulse. (**d**–**f**) Effect of irradiance on (**d**) t_peak_, (**e**) t_off_, and (**f**) adaptation ratio, ratio of peak and plateau photocurrent in ChRmine on illuminating with 1 s light pulse from different light sources. (**g**–**i**) Effect of pulse width on (**g**) t_peak_, (**h**) t_off_, and (**i**) adaptation ratio in ChRmine at 0.049 mW/mm^2^ from different light sources.

The photocurrent kinetics in different opsins is shown in Fig. 2. The normalized photocurrent in different opsins to compare the turn-off of photocurrent is shown in Fig. 2a. In Fig. 2b, time to achieve peak photocurrent (t_peak_) and time to turn-off the photocurrent by 1/e (t_off_) for different opsins are compared at same irradiance. At 1 mW/mm^2^, the t_peak_ and t_off_ are shortest for PsCatCh2.0 and CatCh, respectively. Further, the study shows the effect of changing light source on t_peak_ and t_off_ (Fig. 2c). On illuminating with different light sources at constant irradiance in ChRmine, pure white light results in the shortest t_peak_, whereas, t_off_ is shortest for LED (Fig. 2c). Further, the effect of irradiance and pulse width on t_peak_, t_off_, and the adaptation ratio in ChRmine on illuminating with different light sources is studied in detail (Fig. 2d–i). As is evident, t_peak_ decreases with irradiance and attains same value for all type of light sources at higher irradiance (Fig. 2d). The longer t_peak_ in opsins at low irradiances significantly changes the photocurrent profile (Supplementary Fig. S3). For fixed irradiances, t_peak_ increases with pulse width until the maximum photocurrent is achieved, and thereafter saturates (Fig. 2g, Supplementary Fig. S3). The photocurrent shows early saturation for broadband light sources (Fig. 2g). The t_off_ increases with the irradiance as well as pulse width, which is consistent with the earlier reported experimental results^29^ (Fig. 2e,h). This is due to increased population in the more stable open-state. The adaptation ratio, i.e., the ratio of plateau and peak photocurrent, decreases with both irradiance and pulse width. It decreases more rapidly for broadband light sources (Fig. 2f,i).

**Figure 3:**
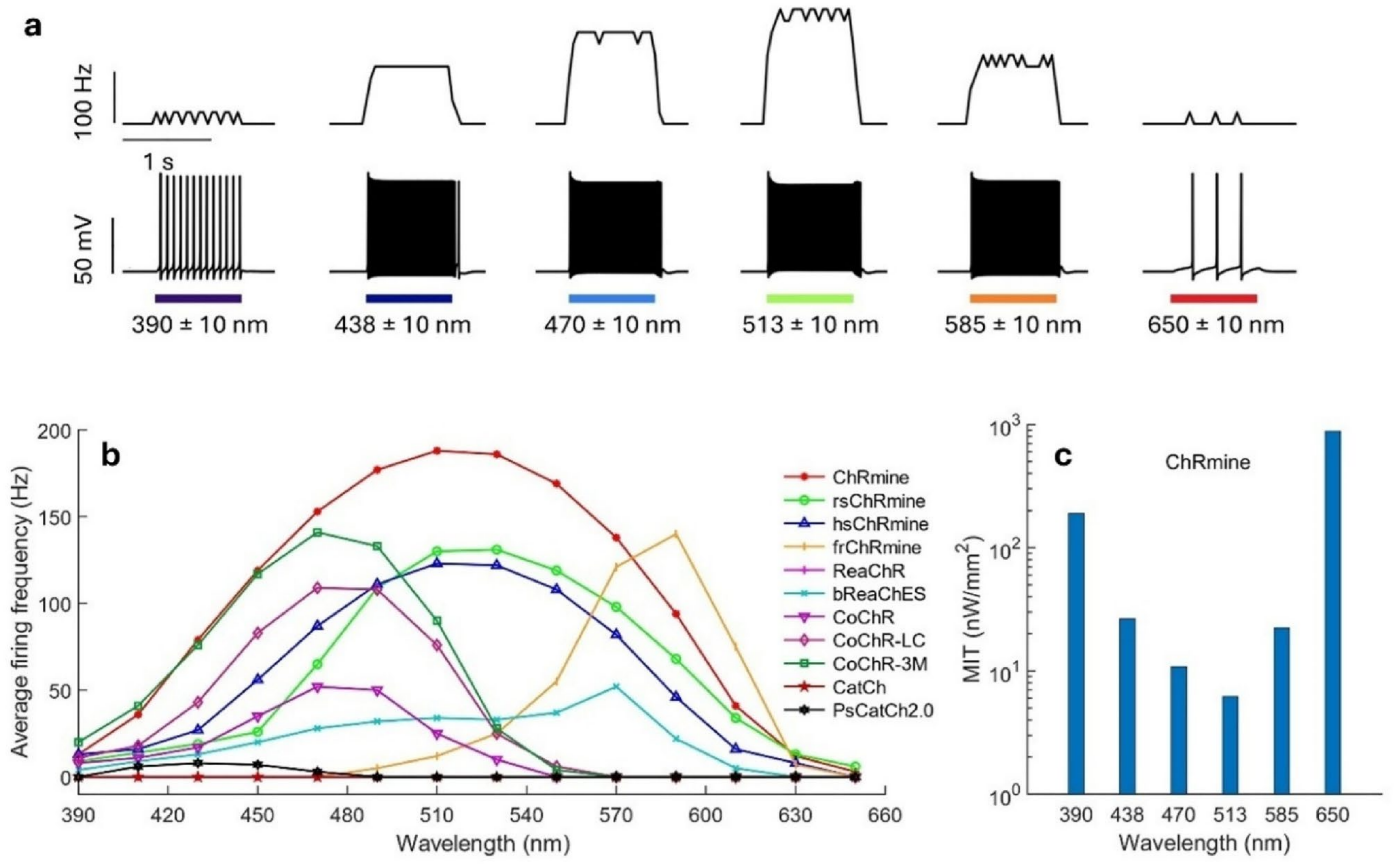
Theoretical simulation of wavelength-sensitivity of optogenetically evoked firing in different opsin-expressing RGNs on illuminating with near monochromatic light sources for 1 s. (**a**) Variation of instantaneous firing rate (upper) and membrane potential (lower) with time on illuminating with LEDs at indicated wavelengths and 1 µW/mm^2^ in ChRmine, and (**b**) corresponding variation of average firing rate in 1 s with wavelength in different opsin-expressing neurons. (**c**) Minimum irradiance threshold (MIT) to evoke spiking at different wavelengths in ChRmine-expressing RGNs.

Color perception is an important feature of normal vision. Figure 3 shows the effect of change in wavelength on the firing response of ChRmine-expressing RGNs. On illuminating with LEDs of different peak wavelengths, the variation of membrane potential and corresponding instantaneous firing rate with time in ChRmine-expressing RGNs is shown in Fig. 3a. Figure 3b shows the variation of average firing rate with wavelength for different opsins. The firing maxima for each opsin is at its peak activation wavelength. The firing rate on either side of peak absorption wavelength decreases at different rates, which is useful to restore color sensitivity in the retina (Fig. 3b). The minimum irradiance threshold (MIT) to evoke single action potential at each wavelength is also determined (Fig. 3c). For ChRmine, MIT is lowest at its peak activation wavelength (513 nm) and highest at 650 nm (Fig. 3c).

**Figure 4:**
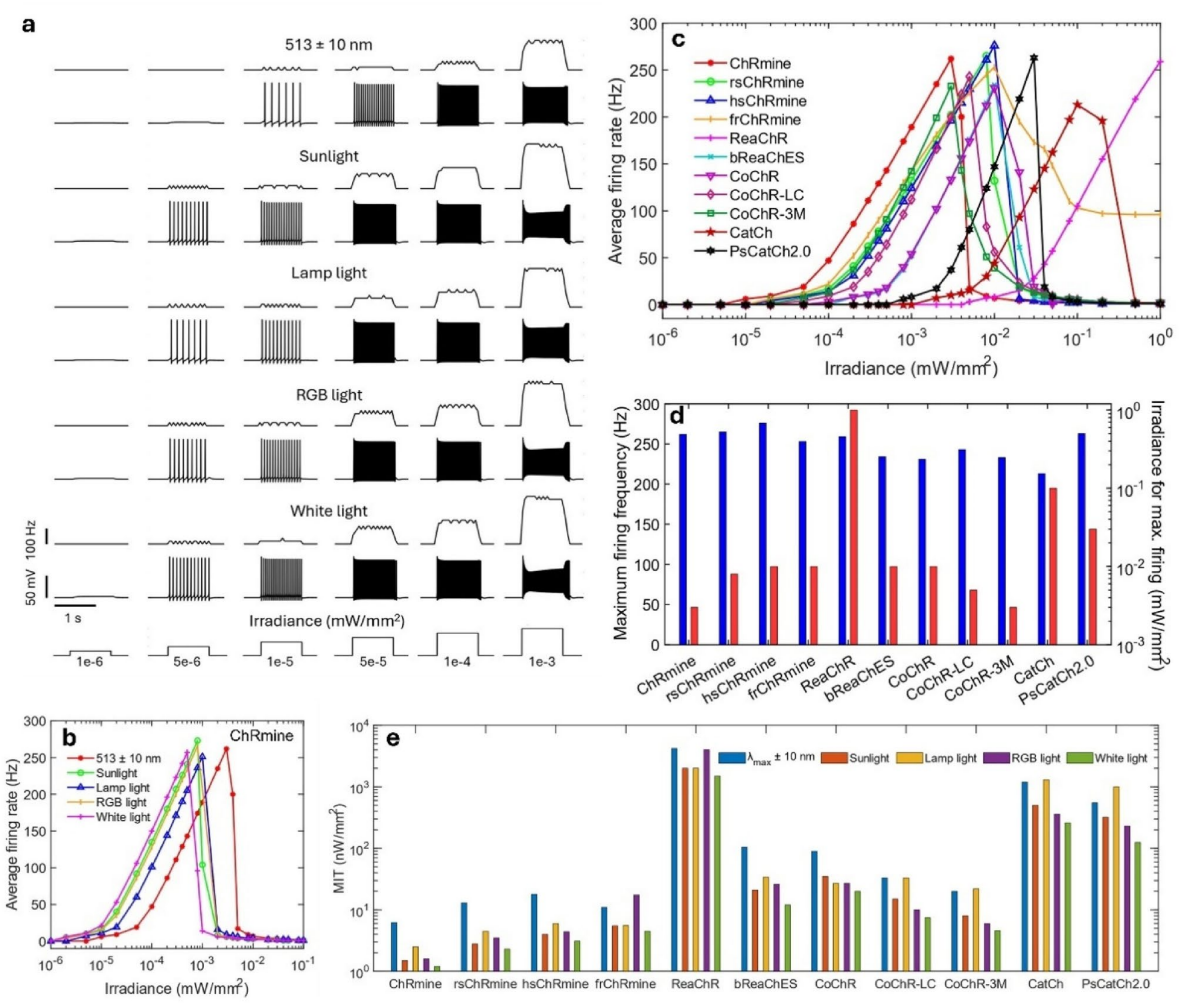
Theoretical simulation of irradiance-dependence of firing response in different opsin-expressing RGNs on optogenetic excitation with near monochromatic and broadband light sources for 1 s light pulse. (**a**) Variation of instantaneous firing rate (upper) and membrane potential (lower) with time on illuminating at indicated irradiances from different light sources, and (**b**) corresponding variation of average firing rate with irradiance in ChRmine-expressing neurons. (**c**) Variation of average firing rate with irradiance in different opsins on illuminating with LEDs, and (**d**) corresponding maximum firing rate, and respective required irradiances with different opsins. (**e**) Minimum irradiance threshold (MIT) to evoke an action potential in different opsins on illuminating with different types of light sources.

The effect of change in irradiance on the firing frequency of different opsin-expressing RGNs is shown in Fig. 4. The spiking patterns in ChRmine-expressing RGNs at different light irradiances show that the broadband light sources evoke firing at an order of magnitude lower irradiance (Fig. 4a). The firing starts at ~ 5 × 10^–6^ mW/mm^2^ for broadband light sources. Figure 4b shows the variation of average firing rate in ChRmine-expressing RGNs with irradiance for different light sources. Although the maximum achievable firing rate in ChRmine-expressing RGNs (~ 250 Hz) is similar for all the light sources, it can be achieved at the lowest irradiance with pure white light, which is an order of magnitude lower than LEDs (Fig. 4b). The firing rate increases from 0 to 250 Hz on changing the irradiance by two orders of magnitude (from 10^–5^ to 10^–4^ mW/mm^2^) using broadband light source, whereas a change of three orders of magnitude (from 10^–5^ to 10^–3^ mW/mm^2^) is needed with LED (Fig. 4b). Therefore, the firing rate in RGNs has better contrast with broadband light sources than LEDs. Although both wavelength and irradiance can change the firing rate, an additional detector would be needed to identify whether the cause due to wavelength or irradiance. On illuminating with LEDs and other light sources, the variation of average firing rate with irradiance in different opsins is shown in Fig. 4c and Supplementary Fig. S4. As is evident, all the opsins attain the maximum firing rate in the range 200–250 Hz, although the required irradiance to achieve maximum firing rate is different for each opsin (Fig. 4d). Further, the irradiance for achieving maximum firing rate is determined for each opsin on illuminating with different light sources (Supplementary Fig. S5).

The minimum irradiance required to evoke spikes in ChRmine-expressing RGNs on illuminating with 1 s light pulse is shown in Fig. 4e. The pure white light evokes spiking at an order of magnitude lower irradiance than LEDs in all the opsins (Fig. 4e). For sunlight, lamp light and RGB light sources, different opsins exhibit irradiance thresholds minima for different sources, depending on the overlapping of their activation spectrum with the source spectrum, as observed earlier for EPD50 for photocurrent (Figs. 1g, 4e).

**Figure 5:**
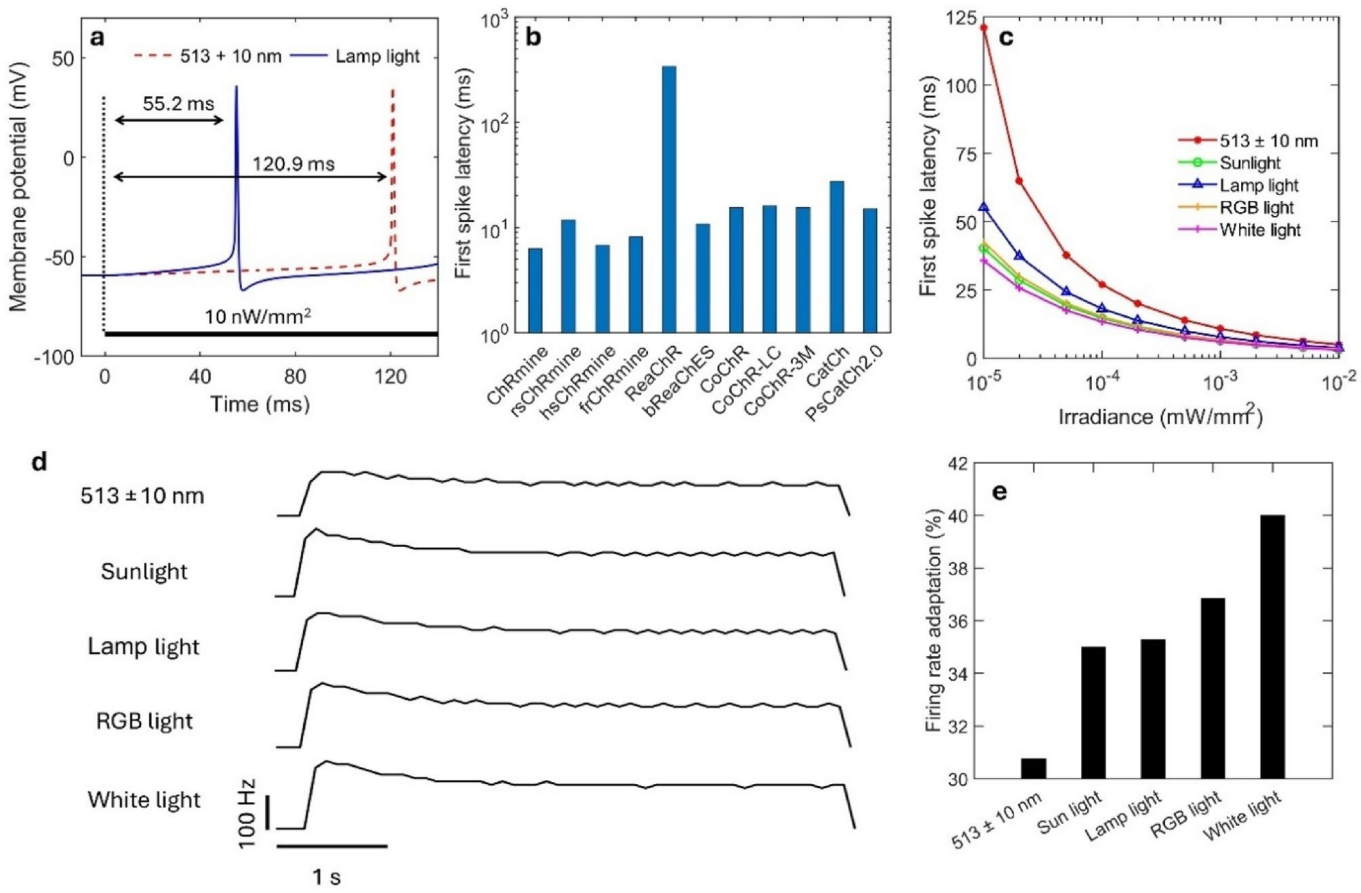
Theoretical simulation of spike latency and firing rate adaptation with time in different opsin-expressing RGNs on illuminating with different light sources. (**a**) First spike latency, i.e., delay in spikes from the light-on, on illuminating with LED and lamp light in ChRmine. (**b**) First spike latency in different opsins on illuminating with LEDs at 5 µW/mm^2^. (**c**) Variation of the first spike latency with irradiance in ChRmine on illuminating with 1 s light pulse from different light sources. (**d**) Variation of instantaneous firing rate with time in ChRmine on illuminating with a 5 s light pulse from different light sources at 0.01 mW/mm^2^ and an order lower expression level, and (**e**) corresponding percentage of firing rate adaptation after 5 s, for different light sources.

Spike latency, i.e., the time duration to get spike after stimulation, is an important factor for visual systems as the inherent delays would lead to mis-localization as things move or change with time^47^. This study shows that, at constant irradiance, changing the light source from LED to lamp light decreases the spike latency from 120.9 to 55.2 ms in ChRmine (Fig. 5a). The first spike latency in different opsin-expressing RGNs is shown in Fig. 5b. At 5 µW/mm^2^, ChRmine and hsChRmine both exhibit very short latency among the studied opsins (Fig. 5b). Further, the effect of irradiance on the latency has been studied in detail (Fig. 5, Supplementary Fig. S6). At lower irradiances, ChRmine-expressing RGNs exhibit shorter spike latencies with broadband light sources in comparison to LED (Fig. 5c). However, at saturating irradiances, shortest latency is achieved for each opsin, which is same for all types of light sources (Supplementary Fig. S6).

The photocurrent in all the opsins adapts with time under sustained illumination and reaches a lower plateau current, which subsequently results in a decrease in the firing rate of neurons. The variation of instantaneous firing rate in ChRmine with time on illuminating with different light sources is shown in Fig. 5d. The percentage of adaptation in the firing rate in 5 s is highest with pure white light (Fig. 5e).

**Figure 6:**
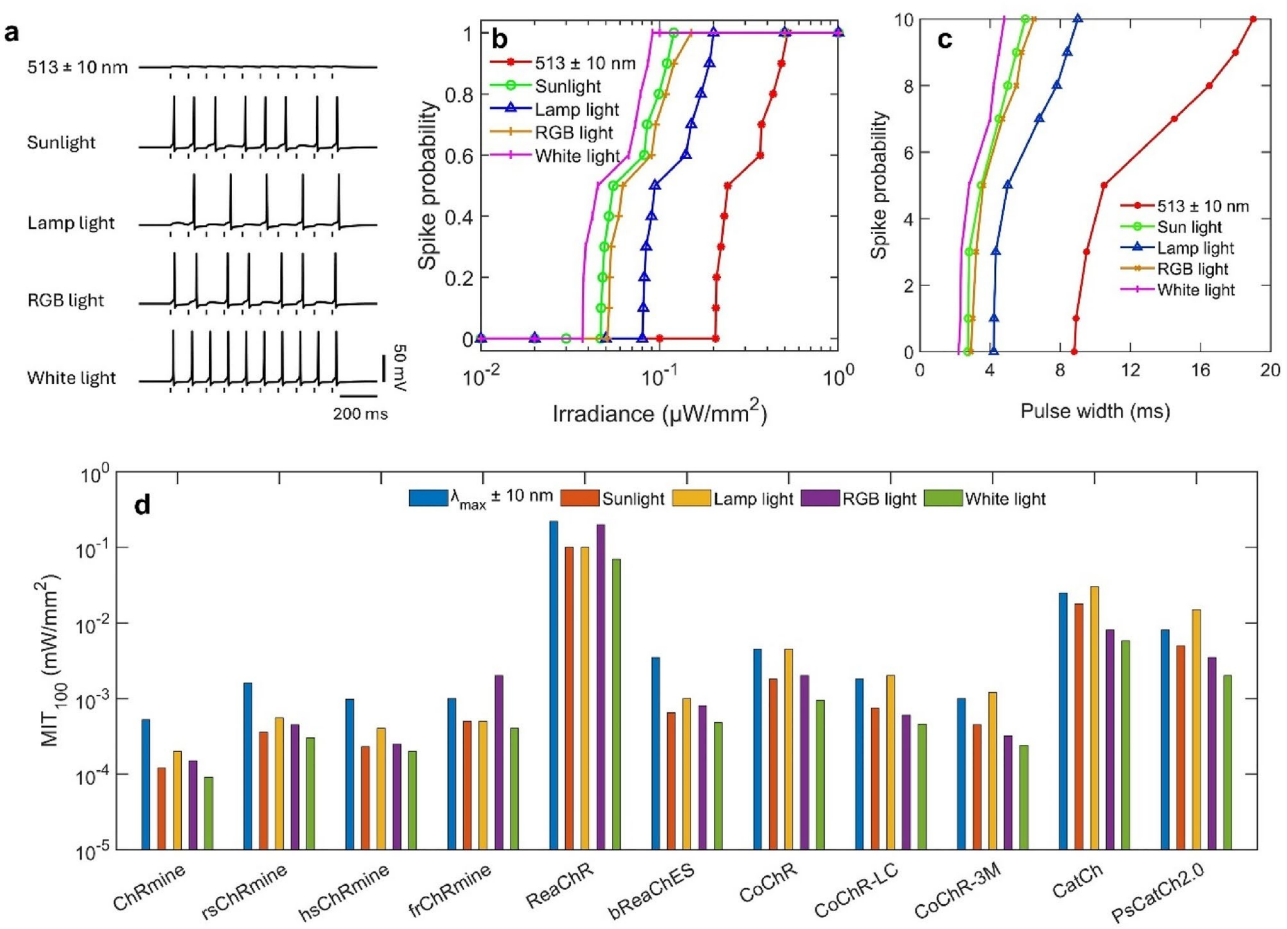
Theoretical simulation of spiking in different opsin-expressing RGNs under pulsed stimulation at 10 Hz on illuminating with different light-sources. (**a**) Variation of membrane potential with time in ChRmine-expressing neurons on illuminating with 5 ms light pulses at 10 Hz from different light sources at 0.1 µW/mm^2^, and (**b**, **c**) corresponding variation of spike probability with (**b**) irradiance at fixed pulse width of 5 ms, and (**c**) pulse width at fixed irradiance of 0.1 µW/mm^2^. (**d**) Minimum irradiance threshold to achieve 100% spiking (MIT_100_) for 5 ms light pulse at 10 Hz in different opsins on illuminating with different light sources.

Generation of temporally precise and high-fidelity optogenetic spiking patterns optogenetics is required for encoding information in the spike sequence. The spiking responses of ChRmine-expressing neurons under pulsed illumination at 10 Hz are shown in Fig. 6a. The effect of irradiance and pulse width on spike probability is shown in Figs. 6b,c. As is evident, on illuminating with pure white light, pulses of an order of lower intensity or shorter duration can evoke temporally precise spike-train in ChRmine-expressing neurons (Fig. 6b,c). The minimum light irradiance to evoke 100% spikes in ChRmine-expressing RGNs at pulses of 5 ms is 0.52 µW/mm^2^ for LED, 0.091 µW/mm^2^ for white light, 0.12 µW/mm^2^ for sunlight, 0.15 µW/mm^2^ for RGB light and 0.2 µW/mm^2^ for lamp light (Fig. 6b). On the other hand, a minimum pulse width of 19 ms for LEDs, 4.8 ms for white light, 6 ms for sunlight, 6.5 ms for RGB light, and 9 ms for lamp light is required to evoke 100% spikes at 0.1 µW/mm^2^ (Fig. 6c). The minimum irradiance threshold to achieve 100% spiking under pulsed stimulation of RGNs expressed with different opsins is shown in Fig. 6d. Broadband light again exhibits an order of magnitude improved performance than LEDs for all the opsins (Fig. 6d).

**Figure 7:**
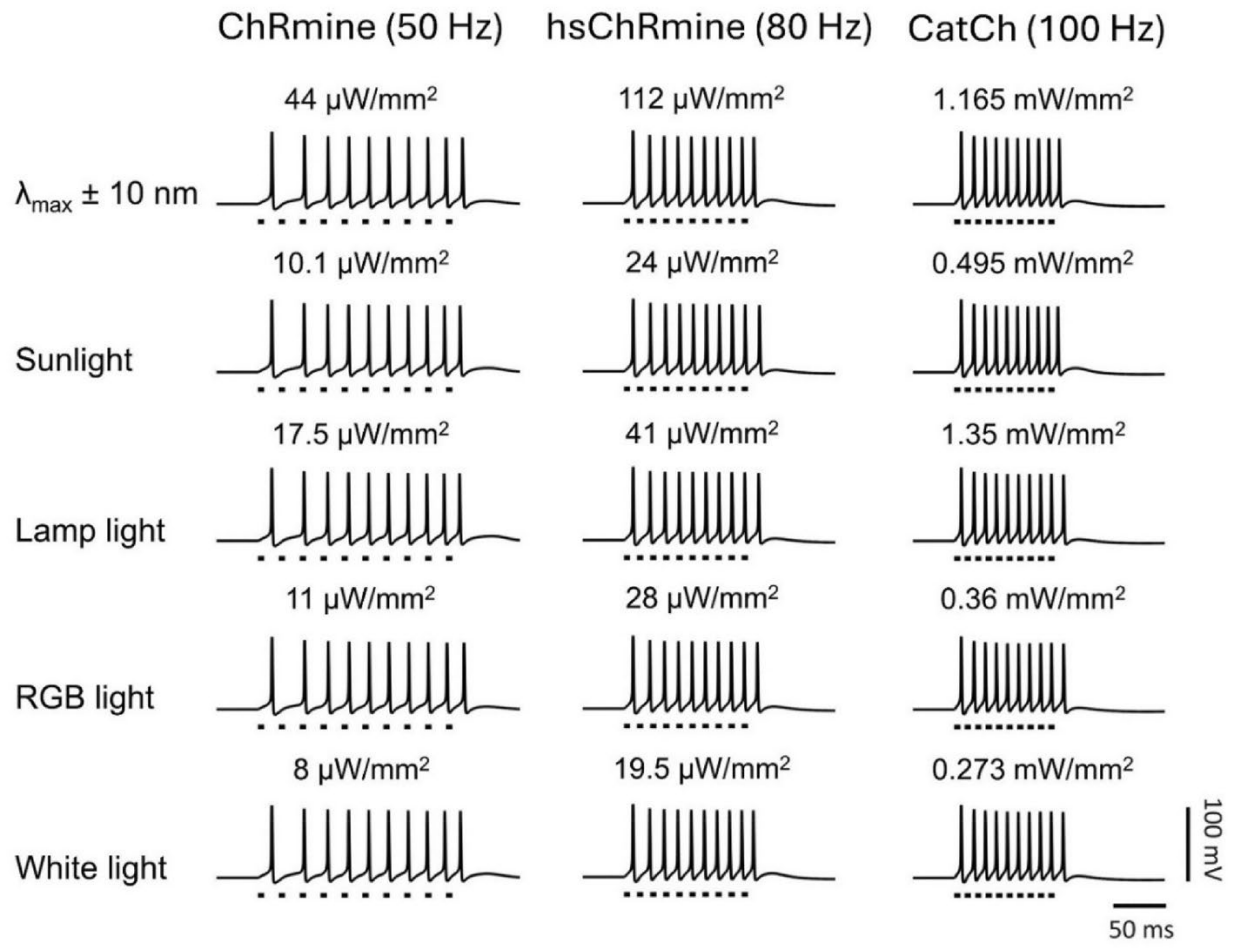
Theoretical simulation of high-frequency optogenetic excitation of different opsin-expressing RGNs on illuminating with different light sources. Variation of membrane potential with time on illuminating with 0.2 ms light pulses at indicated frequencies and irradiances.

The high-frequency limit of temporally precise spikes in optogenetics is governed by the opsin photocurrent off kinetics. As shown in Fig. 2b, the photocurrent in different opsins exhibits different turn-off kinetics. Hence, a detailed analysis has been undertaken to determine the high-frequency limit of the opsins. The study shows that, at a good set of photostimulation conditions, the high-frequency limit for ChRmine, hsChRmine, and CatCh is 50 Hz, 80 Hz, and 100 Hz for all types of light sources, which is sufficient for optogenetic prostheses, as the required firing rate is ~ 50 Hz (Fig. 7).

## Discussion

The present study shows that broadband activation of opsins having broadband activation spectrum allows ultra-low intensity activation of RGNs. ChRmine allows firing at the lowest light intensity among the opsins considered. The minimum intensity required to restore sensation in ChRmine-expressing RGNs is 1.2 nW/mm^2^ (~ 1.96 × 10^10^ photons mm^−2^ s^−1^) for white light and 1.5 nW/mm^2^ (~ 1.87 × 10^10^ photons mm^−2^ s^−1^) for sunlight (Fig. 4). Other opsins that include rsChRmine, hsChRmine, frChRmine, and CoChR-3M also exhibit very low threshold for activation of RGNs (Fig. 4e). The estimated photon flux density is an order of magnitude lower than recently reported results at the lowest intensity to date, *i.e.,* 0.04 µW/mm^2^ (~ 1.03 × 10^11^ photons mm^−2^ s^−1^ at 505 nm from LED) with ex3mV1Co, and 2–3 orders of magnitude lower than reported experiments with other opsins^15–19^. It is reported that sunlight intensity on earth ranges from 440 lx (3.5 µW/mm^2^) to 93,500 lx (0.75 mW/mm^2^) in open playground^48,49^. Ambient sunlight ranges from 0.24 to 0.23 mW/mm^2^ in rooms with multiple large windows, and 0.6–0.12 mW/mm^2^ under a big tree^48,49^. The natural light level at night ranges from 0.08 to 0.8 nW/mm^2^^49,50^. The threshold intensity for broadband activation of RGNs expressed with ChRmine and its mutants is below the above ambient daylight intensities (Fig. 4). Therefore, it can overcome the necessity of extraocular stimulation devices for vision restoration if ChRmine is expressed in the upper layer of retinal neurons^51,52^.

For enabling white light activation, initial experiments reported in the literature were made by co-expressing blue (ChR2), green (C1V1), and red (ReaChR) light sensitive opsins into the targeted neurons that reported a minimum white light intensity ~ 0.06 mW/mm^2^ from halogen lamp is required to generate ~ 100 pA photocurrent, sufficient for neuron activation^26,27,29^. Although the light intensity is much improved, the expression of three opsins is challenging for the available delivery methods. In the present study, the irradiance threshold for evoking action potential in ChRmine-expressing RGNs using the same lamp is 3–4 orders of magnitude lower than the reported white light optogenetic experiments (Fig. 4e)^27,29,30^. More recently, an implantable thin-film based light display consisting of LEDs having 20 nm bandwidth has been designed for optogenetic retinal prostheses, which has shown much improved light levels with ChRmine in human RGNs^53^. The present simulated results predict that the intensity thresholds can be further improved with sunlight and pure white light (Fig. 4).

The photoresponse of different opsin-expressing RGNs is compared on illuminating with different broadband light sources. As observed, the required light intensities with pure white light and sunlight are almost similar for red-shifted opsins, as their absorption maxima overlaps with the spectral peak of sunlight spectrum (Fig. 1c,d and Fig. 3). Moreover, the lamp source in the present study has red-shifted spectral peak, which can be modified for better spectral overlap with the targeted opsin, that would also reduce the cost associated with monochromatic light sources (Fig. 1d). The RGB light source has broad spectrum in the blue-green region, thereby providing lower irradiance threshold for blue-light activated opsins than sunlight and lamp light. The spectral intensity at different wavelengths of RGB light source can be changed by changing the relative intensities of the red, green, and blue LEDs (Fig. 1d). Although lamp light, sunlight and RGB light resulted in improved irradiance thresholds, ideal pure white light source with flat spectrum always gives excitation at the lowest irradiance in all the opsins (Fig. 4e). At present, efforts are going on to develop white light sources exhibiting almost constant spectral intensity over a wide spectrum due to their increasing applications in display and lighting devices^54,55^.

The activation spectrum for ChRmine and its mutants under wide-field illumination has been reported only in the range 390–650 nm, which appears to monotonically decrease above 650 nm^35^. Furthermore, the activation spectrum in the infrared region (800–1300 nm) has been reported for two-photon excitation, which requires very high intensity light in comparison to wide-field illumination^35^. Therefore, infrared activation of ChRmine at ambient light conditions considered in the present study which is of main concern would not be significant. For safety, the maximum permissible radiant exposure (*MPH*_c_) for duration ≤ 3 × 10^4^ s is reported to be ~ 0.77 mW/mm^2^ (calculated for long duration exposure for 400 nm < λ < 700 nm)^56^. In the present study, the maximum light intensity for ChRmine for continuous (2 µW/mm^2^ for LED and 0.5 µW/mm^2^ for white light) and pulsed stimulation (44 µW/mm^2^ for LED and 8 µW/mm^2^ for white light) is much below the permissible maximum exposure. Hence, it appears that infrared light at such intensities would not cause any considerable effect due to the poor sensitivity of ChRmine to infrared light, although it requires to be studied further experimentally.

In comparison to white opsin designed by fusion of multiple opsins having their spectral peak in blue, green, and red regions, ChRmine and its mutants have broadband activation spectrum^26,29,35^. Therefore, the expression of these opsins would be useful to overcome the challenges associated with the effective and safe expression of multiple opsins^29^. It can be further improved using recently reported femtosecond laser pulse-assisted nonviral expression methods^57^. Broadband activation spectrum of ChRmine and its mutants has also enabled broad range wavelength sensitivity of retinal neurons (Fig. 3). The firing rate decays differently on shifting the illumination wavelength either towards blue or red regions from the peak absorption wavelength (Fig. 3). Therefore, color perception would also be restored in these opsin-expressing RGNs at a constant intensity, which would allow multimodal perception along with sensitivity to light intensity.

In optogenetics, fast opsins need higher irradiance, whereas slow opsins cannot evoke temporally precise spikes, as the kinetics and sensitivity are inversely correlated^14^. For the human retina, it is reported that most of the RGNs fire between 20 and 90 Hz (maximum population fire ~ 50 Hz)^58^. The required frequency for retinal neurons is also compared to the refresh frame rate of cinema, i.e., ~ 30 to 50 Hz^19,20^. The present study shows that ChRmine generates high-fidelity spikes up to 50 Hz, which is sufficient to drive a larger population of RGNs in the human retina (Fig. 7). Therefore, ChRmine would be the best opsin for achieving control at low-power (Fig. 4), although, hsChRmine and CatCh result in enhanced firing rates upto 80 Hz and 100 Hz, respectively, at higher irradiance than ChRmine (Fig. 7).

In the retina, the electrical signal starts from photoreceptor cells and propagates towards RGNs via intermediate retinal neuron layers^6^. These intermediate layers consist of different types of neurons, which process the electrical signal and help in encoding different features in the restored vision^6^. Therefore, the excitation of cells in the outer layers of the retina is beneficial for using natural encoding features in the absence of photoreceptors^6^. However, a recent study comparing restored visual function achieved through optogenetic excitation of ON bipolar and RGNs has shown that targeting RGNs is likely a practical and advantageous strategy due to currently available gene delivery methods^9,59^. Moreover, RGNs have a relatively larger cell surface area ~ 5000 µm^2^, which is advantageous for low-power activation and being easy to target^26,60^. The results presented in the present study are also important for restoring vision in partially degenerated retinas, as the remaining photoreceptor cells saturate on illuminating with high-intense monochromatic light sources in optogenetic retinal prostheses. The use of low-level white light sources would be beneficial to take advantage of the remaining healthy photoreceptor cells in the retina.

The proposed theoretical model of broadband optogenetic activation of different opsins-expressing RGNs accurately simulates the reported photocurrent at different wavelengths. Further, the simulated results with broadband light sources are consistent with reported experimental results with white opsin^29,30^. In the present study, the total conductance is determined from reported experimental results in hippocampal neurons, which have a somal surface area ~ 1000 µm^2^. However, the surface area for RGNs ranges from 1105 to 4047 µm^2 ^^26,60^. Therefore, the irradiance thresholds determined in this study would be lower for large surface RGNs. Additionally, the inclusion of dendritic areas will further reduce the irradiance thresholds^26^.

The recent human clinical study used ChrimsonR, which starts activation at 10^13^ photons mm^−2^ s^−1^^13,22^. Therefore, it utilized light-emitting goggles providing retinal irradiance ranging from 4 × 10^12^ to 4 × 10^14^ photons mm^−2^ s^−1^^22^. The present study shows that the required photon flux with ChRmine is ~ 3 orders of magnitude lower than ChrimsonR with sufficient temporal resolution 1.2 nW/mm^2^ (~ 1.96 × 10^10^ photons mm^−2^ s^−1^) for white light and 1.5 nW/mm^2^ (~ 1.87 × 10^10^ photons mm^−2^ s^−1^) for sunlight. Whereas, hsChRmine with improved temporal resolution also starts activation at ~ 3 order lower irradiance 3.1 nW/mm^2^ (~ 5.06 × 10^10^ photons mm^−2^ s^−1^) for white light and 4 nW/mm^2^ (~ 4.98 × 10^10^ photons mm^−2^ s^−1^) for sunlight (Figs. 4, 7). Hence, ChRmine and hsChRmine would provide improved optogenetic vision restoration in future human clinical studies.

The present study reveals that broadband stimulation significantly improves the irradiance thresholds for all the opsins. The analysis shows that ChRmine enables excitation of RGNs under ambient light conditions alongwith sufficient temporal resolution upto 50 Hz. Furthermore, the mutant hsChRmine allows high-frequency control upto 80 Hz with a little higher irradiance than ChRmine. However, the proposed prediction is theoretical. Therefore, the results need to be validated through in-vivo experiments. The method of broadband activation of opsins would be very useful in general optogenetic experiments as heating and tissue photodamage are crucial issues in optogenetics for neurons in the brain or large tissue^61^. The low-light intensity required in broadband activation of opsins would enable noninvasive deeper excitation of neurons at safe surface light intensities. The present study is also important for noninvasive, goggle-free, low-cost optogenetic retinal prostheses.

## Methods

### Model of light-induced ionic current through light-sensitive protein channels

In optogenetics, light-sensitive microbial proteins act as light-gated ion-channels, and therefore allow optical control of neuron membrane potential^5^. These proteins sense light with the help of embedded retinal chromophores in their structure^23^. On illumination with light, the retinal chromophore molecule undergoes photoisomerization at ultrafast (~ fs) time scale and triggers the protein photocycle^23,62,63^. In ChRs, the photocycle generally consists of two or more open-states and a few non-conducting intermediate states besides the ground state^23,37^. On reaching an open-state, the protein channel opens, ions flow across the membrane due to natural ionic concentration and potential difference across the neuron membrane. The rate of flow of ions across the membrane depends on the instantaneous population density of opsin molecules in the open-states, membrane potential ($V_{m}$), unitary conductance of opsin channel, and its expression density over neuron membrane surface^37,39,64^. The population of opsin molecules in the open-states depends on the effective photon flux density ($\phi$), and time ($t$). The photocurrent through the ChRmine molecules embedded within the neuron membrane can be expressed as,

1 $$I_{Opsin} = g_{Opsin} f\left( {\phi , t} \right)\left( {V_{m} - E_{Opsin} } \right)$$

where $g_{Opsin}$ is the total conductance, accounting for both unitary conductance and opsin expression density, and $f\left( {\phi , t} \right)$ accounts for the instantaneous population of opsin molecules in the open-states. $E_{Opsin}$ is the reversal potential of the opsin ion-channel. The total effective photon flux density for broadband light sources has been expressed as,

2 $$\phi = \mathop \sum \limits_{\lambda } \varepsilon_{\lambda } I_{\lambda } \lambda /hc$$

where $\varepsilon_{\lambda }$ is a coefficient that accounts for wavelength-dependent quantum efficiency, $I_{\lambda }$ is light intensity at wavelength $\lambda$, $h$ is Planck’s constant, and $c$ is the speed of light^39,64^. $\varepsilon_{\lambda }$ has been determined by fitting the reported experimental photocurrent in opsin at different wavelengths. The function used for fitting wavelength dependance of $\varepsilon_{\lambda }$ is expressed as,

3 $$\varepsilon_{\lambda } = A_{1} \exp \left( {\frac{{\left( {\lambda - B_{1} } \right)^{2} }}{{2*\left( {\sigma_{1} } \right)^{2} }}} \right) + A_{2} \exp \left( {\frac{{\left( {\lambda - B_{2} } \right)^{2} }}{{2*\left( {\sigma_{2} } \right)^{2} }}} \right) + A_{3} \exp \left( {\frac{{\left( {\lambda - B_{3} } \right)^{2} }}{{2*\left( {\sigma_{3} } \right)^{2} }}} \right)$$

$A_{1} , A_{2} , A_{3, } B_{1} , B_{2} , B_{3} ,\sigma_{1} , \sigma_{2}$, and $\sigma_{3}$ are the fitting constants given in Table 1.

**Table 1:** Parameters for photocurrent model of different opsins^15,17,20,28,32,35,45,66,67^.

Parameter	ChRmine	rsChRmine	hsChRmine	frChRmine	ReaChR	bReaChES	CoChR	CoChR-LC	CoChR-3 M	CatCh	PsCatch2.0	Unit
$G_{d1}$	0.02	0.00625	0.04	0.0167	0.0077	0.025	0.00893	0.00286	0.00118	0.0625	0.05	ms^−1^
$G_{d2}$	0.0133	0.00435	0.02	0.00833	0.00125	0.01	0.00667	0.000833	0.000692	0.01	0.004	ms^−1^
$G_{r}$	5.9E−4	5.9E−4	5.9E−4	5.9E−4	3.3E−5	3.3E−5	5E−4	5E−4	5E−4	2E−4	5E−4	ms^−1^
$\phi_{m}$	2.10e + 15	5.0e + 17	4.0e + 15	1.00e + 15	5.0e + 17	6.0e + 15	4e + 15	3.8e + 15	3.5e + 15	1.5e + 17	3.5e + 16	ph. mm^−2^ s^−1^
$k_{1}$	0.2	4.1	0.22	0.2	1.2	0.4	0.15	0.15	0.15	3	7	ms^−1^
$k_{2}$	0.004	0.1	4E−5	0.004	0.01	0.01	0.01	0.01	0.01	0.1	8	ms^−1^
$k_{f}$	0.001	0.01	0.001	0.001	0.012	0.01	0.008	0.003	0.001	0.03	1	ms^−1^
$k_{b}$	0	0	0	0	0.001	0.04	0.012	0.01	0.01	0.06	0.1	ms^−1^
$G_{f0}$	0.003	0.0032	0.0042	0.003	0.0005	0.002	0.002	0.0005	0.0005	0.02	0.001	ms^−1^
$G_{b0}$	0.005	0.006	0.005	0.005	0.0005	0.002	0.0005	0.001	0.0001	0.039	0.0003	ms^−1^
*p*	0.8	0.8	0.8	0.8	1	1	0.98	0.98	0.98	1	1.2	–
*q*	1	0.1	1	1	1	1	0.98	0.98	0.98	1	1.2	–
$\gamma$	0.05	0.05	0.05	0.05	0.05	0.05	0.05	0.05	0.05	0.05	0.2	–
$E_{Opsin}$	5.64	5.64	5.64	5.64	7	10	25	25	25	− 20	12.5	mV
$g_{Opsin}$	41.14	35.14	53.54	11.28	8.15	38.48	25.47	21.87	21.65	63	43.95	nS
$A_{1}$	0.973	1.058	1	0.958	0.917	0.566	0.894	0.976	0.922	1.245	1.086	–
$A_{2}$	0.0973	− 0.423	0.1	0.134	0.138	0.828	0.304	0.156	0.203	− 0.5	− 0.33	–
$A_{3}$	0	0	0	0	0	0.349	0	0	0	0	0	–
$B_{1}$	515	510	515	585	590	575	480	478	476	478	442	nm
$B_{2}$	450	465	390	545	510	480	420	420	420	509	470	nm
$B_{3}$	0	0	0	0	0	550	0	0	0	0	0	nm
$\sigma_{1}$	42	50	44	16	16	15	30	30	28	40	40	nm
$\sigma_{2}$	40	25	40	25	80	52	40	40	40	31	20	nm
$\sigma_{3}$	0	0	0	0	0	30	0	0	0	0	0	nm

The experimental photocurrent in ChRs exhibits biexponential decay kinetics, indicating the presence of two open-states with different lifetimes. Here, a 4-state photocycle model for ChR has been considered, which consists of two-closed ($C_{1}$ and $C_{2}$) and two-open ($O_{1}$ and $O_{2}$) states^39,45,64^. In dark, the ChR molecule rests in closed state-$C_{1}$, which switches from state $C_{1}$ to $O_{1}$ on illuminating with light. From open state $O_{1}$, molecules either switch to the second open state $O_{2}$ or decay back to $C_{1}$. The $O_{2}$ state is less conductive but has a longer lifetime than $O_{1}$. From $O_{2}$ state, it either switches to the $O_{1}$ state or decays to the $C_{1}$ state. The reversible transition takes place between $O_{1}$ and $O_{2}$ states, which can be both due to thermal relaxation and light-induced. From $C_{2}$, it can be either photo-excited back to $O_{2}$ or thermally relax to $C_{1}$. The rate of switching of molecules from $C_{2}$ to $C_{1}$ is much slower than other rate constants^40,41^.

Considering, $C_{1}$, $O_{1}$, $C_{2}$, and $O_{2}$ to denote the instantaneous fraction of ChR molecules in each of the four states such that $C_{1} + O_{1} + O_{2} + C_{2} = 1,$ the rate of change of populations can be described by the following differential equations,

4 $$\dot{C}_{1} = G_{d1} O_{1} - G_{a1} \left( \phi \right)C_{1} + G_{r} C_{2}$$

5 $$\dot{O}_{1} = G_{a1} \left( \phi \right)C_{1} - \left( {G_{d1} + G_{f} \left( \phi \right)} \right)O_{1} + G_{b} \left( \phi \right)O_{2}$$

6 $$\dot{O}_{2} = G_{a2} \left( \phi \right)C_{2} - \left( {G_{d2} + G_{b} \left( \phi \right)} \right)O_{2} + G_{f} \left( \phi \right)O_{1}$$

7 $$\dot{C}_{2} = G_{a2} \left( \phi \right)O_{2} - \left( {G_{a2} \left( \phi \right) + G_{r} } \right)C_{2}$$

where, $G_{a1} , G_{a2} , G_{d1} , G_{d2}$, $G_{f} ,G_{b}$ and $G_{r}$ are the rate constants for transitions $C_{1} \to O_{1} , C_{2} \to O_{2} ,O_{1} \to C_{1} ,O_{2} \to C_{2} ,O_{1} \to O_{2} ,O_{2} \to O_{1}$ and $C_{2} \to C_{1}$ respectively. The light-dependent rate functions can be described as $G_{a1} \left( \phi \right) = k_{1} \phi^{p} /(\phi^{p} + \phi_{m}^{p} )$*,*$G_{a2} \left( \phi \right) = k_{2} \phi^{p} /(\phi^{p} + \phi_{m}^{p} )$, $G_{f} \left( \phi \right) = G_{f0} + k_{f} \phi^{q} /(\phi^{q} + \phi_{m}^{q} )$*,* and $G_{b} \left( \phi \right) = G_{b0} + k_{b} \phi^{q} /(\phi^{q} + \phi_{m}^{q} )$^41,64,65^. Since there are two open states in the 4-state model, $f_{\phi } \left( {\phi , t} \right) = O_{1} + \gamma O_{2}$, where*,* $\gamma$ is the ratio of conductance of the open states. The model parameters for the photocurrent of ChRs have been taken from reported experimental and theoretical results (Table 1).

### Model of optogenetic control in ChR-expressing retinal ganglion neurons

RGNs form the only pathway by which the retina communicates with the brain^68^. The RGNs receive signals from photoreceptor cells via intermediate circuitry. Direct optogenetic excitation of RGNs has been reported feasible in different experiments, including the recent clinical human trial^22^. The mathematical model of the optogenetic excitation of ChR-expressing RGNs has been formulated by integrating the photocurrent through ChR with the ionic currents in the well-established biophysical circuit model of RGNs by Fohlmeister and Miller^45,69,70^. Therefore, the rate of change of membrane potential in the ChR-expressing RGNs depends on five nonlinear natural ionic currents and an opsin-mediated photocurrent as follows,

8 $$C_{m} \dot{V_{m}} = { } - \left( {I_{Na} + I_{K} + I_{KA} + I_{Ca } + I_{KCa} + I_{L} } \right) + I_{Opsin}$$

where, $C_{m}$ is the membrane capacitance of RGNs ($C_{m}$ = 1 µF/cm^2^)^45,69,70^. $I_{Na}$ is the sodium current, $I_{K}$ and $I_{KA}$ are the different potassium currents, $I_{Ca}$ is calcium current and $I_{L}$ is the leakage current. Each of these currents can be described as, $I_{f} = g_{f} m^{p} h^{q} \left( {V_{m} - E_{f} } \right)$*,* where $g_{f}$ is maximal conductance, $m$ is the activation variable (with exponent $p$), $h$ is the inactivation variable (with exponent $q$), and $E_{f}$ is the reversal potential, except $I_{KCa}$ and $I_{L}$^69,70^. The kinetics of each of the gating functions $x$ ($m$ or $h$) depends on the voltage-dependent gating functions of each ion channel ($\alpha_{x}$ and $\beta_{x} )$ as described in Table 2^45,69,70^, and obeys the first-order kinetics as,

9 $$\dot{x} = - \left( {\alpha_{x} + \beta_{x} } \right) x + \alpha_{x}$$

**Table 2:** Gating function parameters of ion-channels for retinal ganglion neurons^45,69,70^.

$I_{ionic}$	Gating variable	$\alpha$	$\beta$
$I_{Na}$	$p = 3$	$\frac{{ - 0.6\left( {V_{m} + 30} \right)}}{{\exp \left[ { - 0.1\left( {V_{m} + 30} \right)} \right] - 1}}$	$20 {\text{exp}}\left[ { - 0.055\left( {V_{m} + 55} \right)} \right]$
	$q = 1$	$0.4 {\text{exp}}\left[ { - 0.05\left( {V_{m} + 50} \right)} \right]$	$\frac{6}{{\exp \left[ { - 0.1\left( {V_{m} + 20} \right)} \right] + 1}}$
$I_{K}$	$p = 4$	$\frac{{ - 0.02 \left( {V_{m} + 40} \right)}}{{\exp \left[ { - 0.1\left( {V_{m} + 40} \right)} \right] - 1}}$	$0.4{\text{ exp}}\left[ { - 0.0125\left( {V_{m} + 50} \right)} \right]$
$I_{Ca}$	$p = 3$	$\frac{{ - 0.3 \left( {V_{m} + 13} \right)}}{{\exp \left[ { - 0.1\left( {V_{m} + 13} \right)} \right] - 1}}$	$10 {\text{exp}}\left[ { - 0.055\left( {V_{m} + 38} \right)} \right]$
$I_{KA}$	$p = 3$	$\frac{{ - 0.006\left( {V_{m} + 90} \right)}}{{\exp \left[ {0.1\left( {V_{m} + 90} \right)} \right] - 1}}$	$0.1\exp \left[ {0.1\left( {V_{m} + 30} \right)} \right]$
	$q = 1$	$0.04 {\text{exp}}\left[ {0.05\left( {V_{m} + 40} \right)} \right]$	$\frac{0.6}{{1 + \exp \left[ {0.1\left( {V_{m} + 40} \right)} \right]}}$

The currents $I_{L}$ and $I_{KCa}$ can be described as follows,

10 $$I_{L} = g_{L} \left( {V_{m} - E_{L} } \right)$$

11 $$I_{KCa} = g_{KCa } \left( {V_{m} - E_{Ca} } \right)$$

where $E_{Ca}$ changes with concentration of calcium and can be modeled using the Nernst equation as,

12 $$E_{Ca} = \frac{RT}{{2F}} ln\left[ {\frac{{\left[ {Ca^{2 + } } \right]_{e} }}{{\left[ {Ca^{2 + } } \right]_{i} \left( t \right)}}} \right]$$

where, $[Ca^{2 + } ]_{e}$ is extracellular Ca^2+^ concentration ($[Ca^{2 + } ]_{e}$ = 1.8 mM), $\left[ {Ca^{2 + } } \right]_{i}$ is intracellular concentration with initial value 10^–4^ mM, $F$ is Faraday constant, $R$ is gas constant, and $T$ is temperature ($T =$ 295 K)^45,69,70^.

The rate of change in $\left[ {Ca^{2 + } } \right]_{i}$ is expressed as,

13 $$[\mathop {Ca^{2 + } }\limits^{ \cdot } ]_{i} = \frac{{ - 3 I_{Ca} }}{2Fr} - \frac{{\left( {[Ca^{2 + } ]_{i} - [Ca^{2 + } ]_{res} } \right)}}{{\tau_{Ca} }}$$

where $[Ca^{2 + } ]_{res}$ is residual level concentration ($[Ca^{2 + } ]_{res}$ = 10^–4^ mM), $\tau_{Ca}$ is calcium removal time ($\tau_{Ca}$ = 50 ms), and $3/\left( {2Fr} \right)$ = 0.000015^69^. The $g_{KCa }$ is ligand-gated conductance and varies as,

14 $$g_{KCa } = \overline{g}_{KCa } \frac{{\left( {\left[ {Ca^{2 + } } \right]_{i} /\left[ {Ca^{2 + } } \right]_{diss} } \right)^{2} }}{{1 + \left( {\left[ {Ca^{2 + } } \right]_{i} /\left[ {Ca^{2 + } } \right]_{diss} } \right)^{2} }}$$

where $\left[ {Ca^{2 + } } \right]_{diss}$ is dissociation constant ($\left[ {Ca^{2 + } } \right]_{diss}$ = 10^–3^ mM). The parameters for the model are given in Table 3^45,69,70^.

**Table 3:** Retinal ganglion neuron model parameters^45,69,70^.

Parameter	Value
$g_{Na}$	50 ${\text{mS/cm}}^{2}$
$g_{K}$	12 ${\text{mS/cm}}^{2}$
$g_{Ca}$	2.2 ${\text{mS/cm}}^{2}$
$g_{KA}$	36 ${\text{mS/cm}}^{2}$
$\overline{g}_{KCa}$	0.05 ${\text{mS/cm}}^{2}$
$g_{L}$	0.147 ${\text{mS/cm}}^{2}$
$E_{Na}$	35 ${\text{mV}}$
$E_{K}$	− 75 ${\text{mV}}$
$E_{L}$	− 61 ${\text{mV}}$

Experimental results with different opsins have been reported in hippocampal neurons and HEK293 cells. The total surface area of the soma of these cells is smaller than ~ 1000 µm^2 ^^27,45^. The photocurrent amplitude has been matched to get the total conductance value for the present model (Table 1). Thus, the conductance per unit area is calculated using 1000 µm^2^ area. Similarly, the conductance per unit area for other opsins has been determined. The optogenetic response of different opsin-expressing RGNs on illuminating with different types of light is studied through numerical simulations using Eqs. (1–14), and gating functions and parameters, given in Tables 1, 2, 3 in MATLAB.

### Supplementary Information

Supplementary Figures.

## Data Availability

All data are presented in the manuscript and figures.

## References

[1] Fleckenstein M (2021). Age-related macular degeneration. Nat. Rev. Disease Primers.

[2] Cehajic-Kapetanovic J, Singh MS, Zrenner E, MacLaren RE (2023). Bioengineering strategies for restoring vision. Nat. Biomed. Eng..

[3] Palanker D (2023). Electronic retinal prostheses. Cold Spring Harb. Perspect Med..

[4] Emiliani V (2022). Optogenetics for light control of biological systems. Nat. Rev. Methods Primers.

[5] Deisseroth K (2015). Optogenetics: 10 years of microbial opsins in neuroscience. Nat. Neurosci..

[6] Pan ZH, Lu Q, Bi A, Dizhoor AM, Abrams GW (2015). Optogenetic approaches to restoring vision. Ann. Rev. Vis. Sci..

[7] Bansal A, Shikha S, Zhang Y (2023). Towards translational optogenetics. *Nat*. Biomed. Eng..

[8] Roska B, Sahel JA (2018). Restoring vision. Nature.

[9] Lindner M, Gilhooley MJ, Hughes S, Hankins MW (2022). Optogenetics for visual restoration: From proof of principle to translational challenges. Prog. Retin. Eye Res..

[10] Yan B (2023). A clinically viable approach to restoring visual function using optogenetic gene therapy. Mol. Ther. Methods Clin. Dev..

[11] Bi A (2006). Ectopic expression of a microbial-type rhodopsin restores visual responses in mice with photoreceptor degeneration. Neuron.

[12] Soltan A (2018). A head mounted device stimulator for optogenetic retinal prosthesis. J. Neural Eng..

[13] McGregor JE (2020). Optogenetic restoration of retinal ganglion cell activity in the living primate. Nat. Commun..

[14] Klapoetke NC (2014). Independent optical excitation of distinct neural populations. Nat. Methods..

[15] Ganjawala TH, Lu Q, Fenner MD, Abrams GW, Pan ZH (2019). Improved CoChR variants restore visual acuity and contrast sensitivity in a mouse model of blindness under ambient light conditions. Mol. Ther..

[16] Chaffiol A (2017). A new promoter allows optogenetic vision restoration with enhanced sensitivity in macaque retina. Mol. Ther..

[17] Chen F (2022). Visual function restoration with a highly sensitive and fast Channelrhodopsin in blind mice. Signal Transduct. Target. Ther..

[18] Too LK (2022). Optogenetic restoration of high sensitivity vision with bReaChES, a red-shifted channelrhodopsin. Sci. Rep..

[19] Bansal H, Roy S, Giudice GL (2023). Recent Advances in Optogenetic Retinal Prostheses. Medical and Surgical Retina: Recent Innovation, New Perspective, and Applications.

[20] Sengupta A (2016). Red-shifted channelrhodopsin stimulation restores light responses in blind mice, macaque retina, and human retina. EMBO Mole. Med..

[21] Gauvain G (2021). Optogenetic therapy: High spatiotemporal resolution and pattern discrimination compatible with vision restoration in non-human primates. Commun. Biol..

[22] Sahel JA (2021). Partial recovery of visual function in a blind patient after optogenetic therapy. Nat. Med..

[23] Schneider F, Grimm C, Hegemann P (2015). Biophysics of channelrhodopsin. Ann. Rev. Biophys..

[24] Chaffiol A (2022). In vivo optogenetic stimulation of the primate retina activates the visual cortex after long-term transduction. Mol. Ther. Methods Clin. Dev..

[25] Watanabe Y (2021). Development of an optogenetic gene sensitive to daylight and its implications in vision restoration. NPJ Regen. Med..

[26] Batabyal S, Cervenka G, Birch D, Kim YT, Mohanty S (2015). Broadband activation by white-opsin lowers intensity threshold for cellular stimulation. Sci. Rep..

[27] Batabyal S, Cervenka G, Ha JH, Kim YT, Mohanty S (2015). Broad-band activatable white-opsin. PLoS One.

[28] Kim CK (2016). Simultaneous fast measurement of circuit dynamics at multiple sites across the mammalian brain. Nat. Methods..

[29] Dhakal K, Batabyal S, Wright W, Kim YT, Mohanty S (2015). Optical delivery of multiple opsin-encoding genes leads to targeted expression and white-light activation. Light Sci. Appl..

[30] Satpathy S (2015). Broad spectral excitation of opsin for enhanced stimulation of cells. Opt. Lett..

[31] Sineshchekov, O. A. *et al*. Conductance mechanisms of rapidly desensitizing cation channelrhodopsins from cryptophyte algae. *mBio* **11**, e00657–20 (2020). 10.1128/mBio.00657-20. 10.1128/mBio.00657-20 PMC7175095 32317325

[32] Marshel JH (2019). Cortical layer–specific critical dynamics triggering perception. Science.

[33] Pyari G, Bansal H, Roy S (2022). Ultra-low power deep sustained optogenetic excitation of human ventricular cardiomyocytes with red-shifted opsins: a computational study. J. Physiol..

[34] Hsueh B (2023). Cardiogenic control of affective behavioural state. Nature.

[35] Kishi KE (2022). Structural basis for channel conduction in the pump-like channelrhodopsin ChRmine. Cell.

[36] Sridharan S (2022). High-performance microbial opsins for spatially and temporally precise perturbations of large neuronal networks. Neuron.

[37] Williams JC (2013). Computational optogenetics: Empirically-derived voltage-and light-sensitive channelrhodopsin-2 model. PLoS Comput. Biol..

[38] Bansal H, Gupta N, Roy S (2020). Theoretical analysis of low-power bidirectional optogenetic control of high-frequency neural codes with single spike resolution. Neuroscience.

[39] Bansal H, Gupta N, Roy S (2020). Comparison of low-power, high-frequency and temporally precise optogenetic inhibition of spiking in NpHR, eNpHR3.0 and Jaws-expressing neurons. Biomed. Phys. Eng. Express.

[40] Bansal H, Pyari G, Roy S (2022). Co-expressing fast channelrhodopsin with step-function opsin overcomes spike failure due to photocurrent desensitization in optogenetics: a theoretical study. J. Neural Eng..

[41] Bansal H, Pyari G, Roy S (2023). Optogenetic generation of neural firing patterns with temporal shaping of light pulses. Photonics.

[42] Saran S, Gupta N, Roy S (2018). Theoretical analysis of low-power fast optogenetic control of firing of Chronos-expressing neurons. Neurophoton.

[43] Gupta N, Bansal H, Roy S (2019). Theoretical optimization of high-frequency optogenetic spiking of red-shifted very fast-Chrimson-expressing neurons. Neurophoton.

[44] Antolik J, Sabatier Q, Galle C, Frégnac Y, Benosman R (2021). Assessment of optogenetically-driven strategies for prosthetic restoration of cortical vision in large-scale neural simulation of V1. Sci. Rep..

[45] Bansal H, Gupta N, Roy S (2021). Theoretical analysis of optogenetic spiking with ChRmine bReaChES and CsChrimson-expressing neurons for retinal prostheses. J. Neural Eng..

[46] Carreres-Prieto D, García JT, Cerdán-Cartagena F, Suardiaz-Muro J (2020). Performing calibration of transmittance by single rgb-led within the visible spectrum. Sensors.

[47] Schlag J, Schlag-Rey M (2002). Through the eye, slowly: Delays and localization errors in the visual system. Nat. Rev. Neurosci..

[48] Bhandary SK, Dhakal R, Sanghavi V, Verkicharla PK (2021). Ambient light level varies with different locations and environmental conditions: Potential to impact myopia. PLoS One.

[49] Walbeek TJ, Harrison EM, Gorman MR, Glickman GL (2021). Naturalistic intensities of light at night: A review of the potent effects of very dim light on circadian responses and considerations for translational research. Front. Neurol..

[50] Michael PR, Johnston DE, Moreno W (2020). A conversion guide: solar irradiance and lux illuminance. J. Meas. Eng..

[51] Berry MH (2019). Restoration of high-sensitivity and adapting vision with a cone opsin. Nat. Commun..

[52] Kralik J, van Wyk M, Stocker N, Kleinlogel S (2022). Bipolar cell targeted optogenetic gene therapy restores parallel retinal signaling and high-level vision in the degenerated retina. Commun. Biol..

[53] Knudsen, E. B. *et al*. A thin-film optogenetic visual prosthesis. *bioRxiv* (2023). 10.1101/2023.01.31.526482

[54] Yang QY, Lehn JM (2014). Bright white-light emission from a single organic compound in the solid State. Angew. Chem. Int. Ed. Engl..

[55] Wang S (2017). A semi-conductive organic–inorganic hybrid emits pure white light with an ultrahigh color rendering index. J. Mater. Chem. C.

[56] Yan B, Vakulenko M, Min SH, Hauswirth WW, Nirenberg S (2016). Maintaining ocular safety with light exposure, focusing on devices for optogenetic stimulation. Vision Res..

[57] Batabyal S, Kim S, Wright W, Mohanty S (2021). Laser-assisted targeted gene delivery to degenerated retina improves retinal function. J. Biophoton..

[58] Reinhard K, Münch TA (2021). Visual properties of human retinal ganglion cells. PLoS One.

[59] Lu Q, Pan ZH (2021). Optogenetic strategies for vision restoration. Adv. Exp. Med. Biol..

[60] Fohlmeister JF, Cohen ED, Newman EA (2010). Mechanisms and distribution of ion channels in retinal ganglion cells: Using temperature as an independent variable. J. Neurophysiol..

[61] Owen SF, Liu MH, Kreitzer AC (2019). Thermal constraints on in vivo optogenetic manipulations. Nat. Neurosci..

[62] Roy S, Singh CP, Reddy KP (2001). Generalized model for all-optical light modulation in bacteriorhodopsin. J. Appl. Phys..

[63] Roy S, Kikukawa T, Sharma P, Kamo N (2006). All-optical switching in *pharaonis phoborhodopsin* protein molecules. IEEE Trans. Nanobiosci..

[64] Evans BD, Jarvis S, Schultz SR, Nikolic K (2016). PyRhO: A multiscale optogenetics simulation platform. Front. Neuroinform..

[65] Pyari G, Bansal H, Roy S (2023). Optogenetically mediated large volume suppression and synchronized excitation of human ventricular cardiomyocytes. Pflügers Arch. Europ. J. Physiol..

[66] Kleinlogel S (2011). Ultra-light-sensitive and fast neuronal activation with the Ca^2+^-permeable channelrhodopsin CatCh. Nat. Neurosci..

[67] Lin JY (2013). ReaChR: a red-shifted variant of channelrhodopsin enables deep transcranial optogenetic excitation. Nat. Neurosci..

[68] D'Souza S, Lang RA (2020). Retinal ganglion cell interactions shape the developing mammalian visual system. Development.

[69] Fohlmeister JF, Coleman PA, Miller RF (1990). Modeling the repetitive firing of retinal ganglion cells. Brain Res..

[70] Fohlmeister JF, Miller RF (1997). Impulse encoding mechanisms of ganglion cells in the tiger salamander retina. J. Neurophysiol..

