# Supplementary material for: Theoretical prediction of broadband ambient light optogenetic vision restoration with ChRmine and its mutants
Source: Sci Rep. 2024 May 21;14:11642. doi: 10.1038/s41598-024-62558-2 (PMC11109128; doi:10.1038/s41598-024-62558-2)
Supplement: Supplementary file 1 — Supplementary Figures. [file 41598_2024_62558_MOESM1_ESM.docx]

**Theoretical Prediction of Broadband Ambient Light Optogenetic Vision Restoration with ChRmine and its Mutants**

Himanshu Bansal, Gur Pyari, and Sukhdev Roy

**Supplementary Figures**


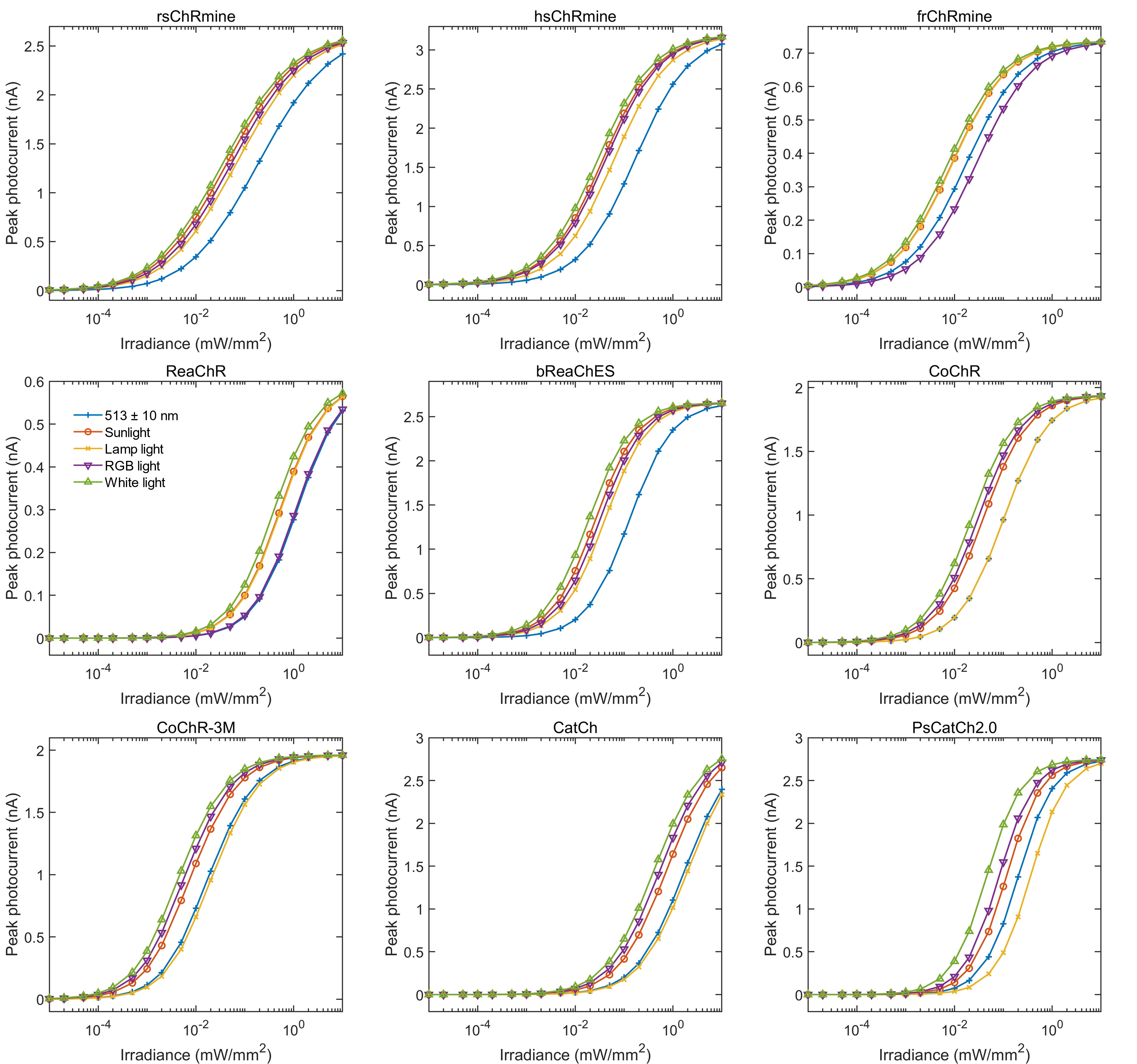


**Supplementary Fig. S1 Theoretical simulation of photocurrent and light-sensitivity in different opsins on illuminating with near monochromatic and broadband light sources. (a)** Variation of peak photocurrent with irradiance in different opsins on illuminating with 1s light pulse.


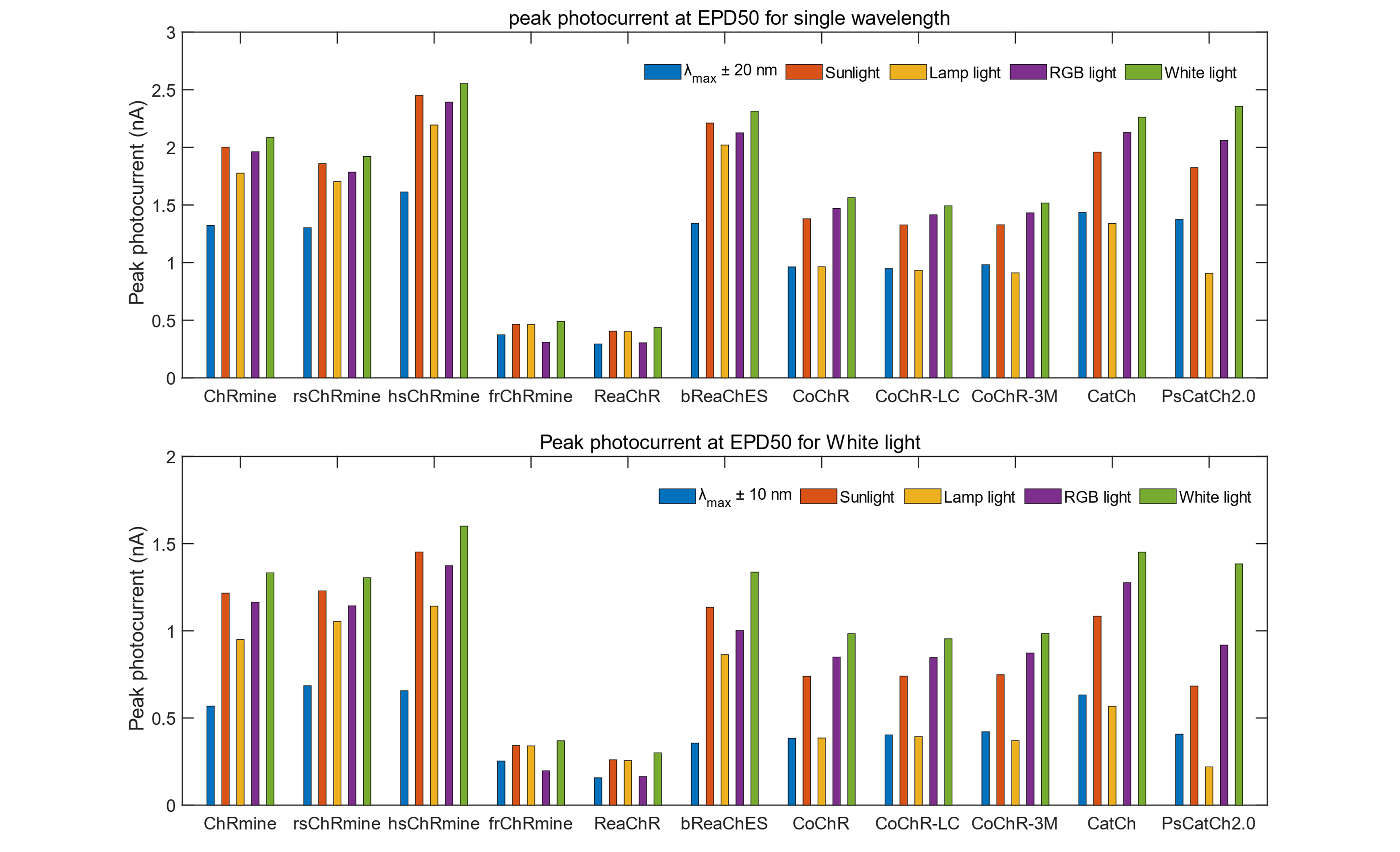


**Supplementary Fig. S2 Theoretical simulation of peak photocurrent amplitude.** Peak photocurrent in opsins on illuminating with different types of light sources for 1s at their respective (upper) EPD50 for monochromatic light source, and (lower) EPD50 for pure white light sources.


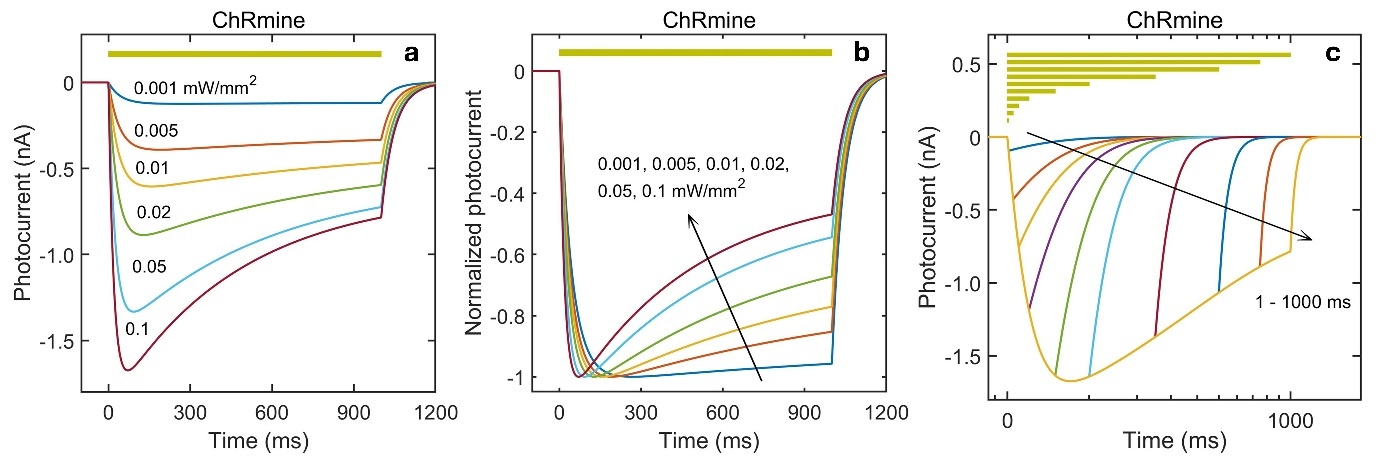


**Supplementary Fig. S3 Effect of irradiance and pulse width on photocurrent temporal profile in ChRmine.** **(a)** Variation of photocurrent with time on illuminating with 1s light pulse at indicated irradiances. **(b)** corresponding variation of normalized photocurrent. **(c)** Variation of photocurrent with time on illuminating with light pulses of different pulse widths at 0.1 mW/mm^2^.


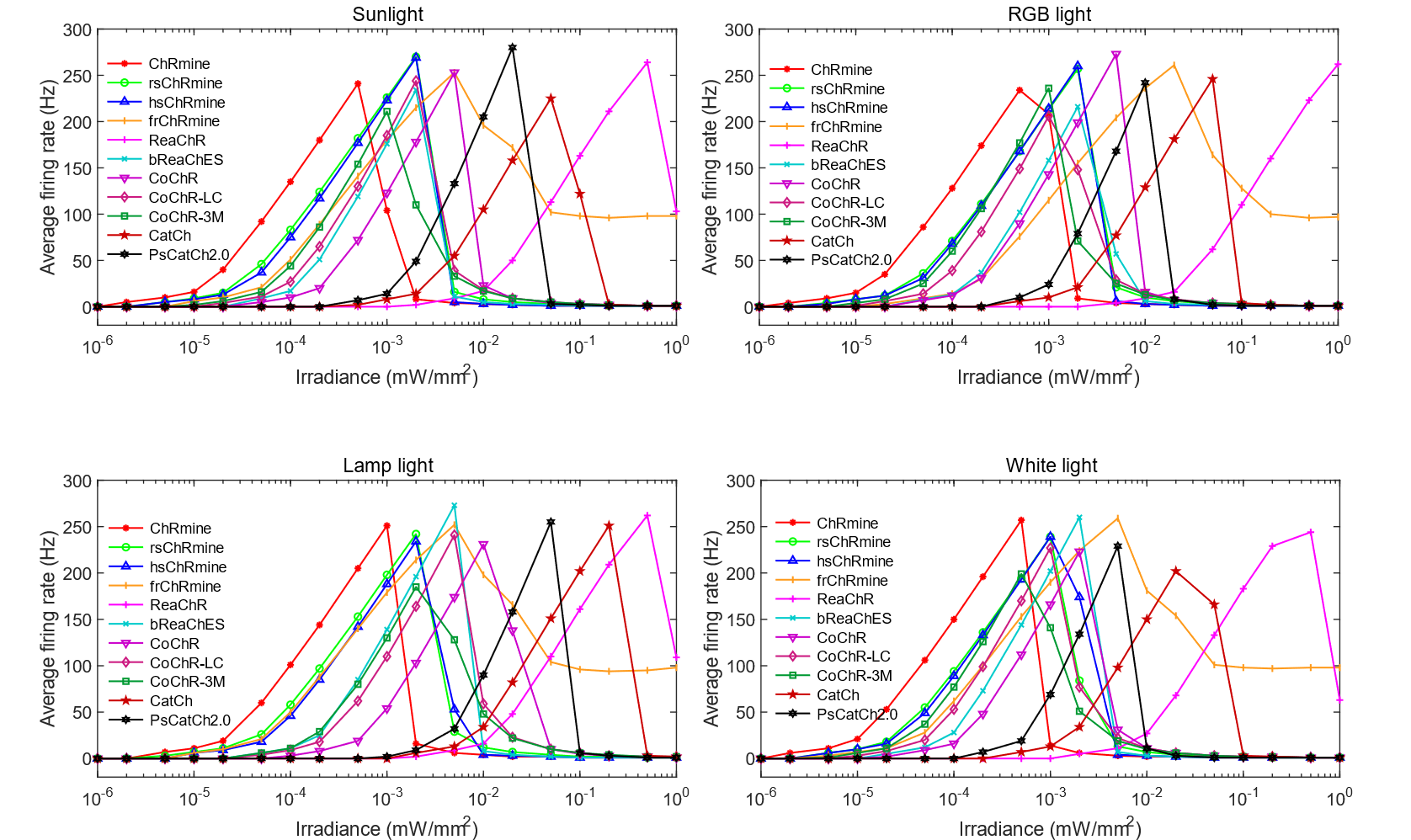


**Supplementary Fig. S4** **Theoretical simulation of irradiance-dependence of firing response in different opsin-expressing RGNs on optogenetic excitation with near monochromatic and broadband light sources.** Variation of average firing rate with irradiance in different opsins using 1s light pulse.


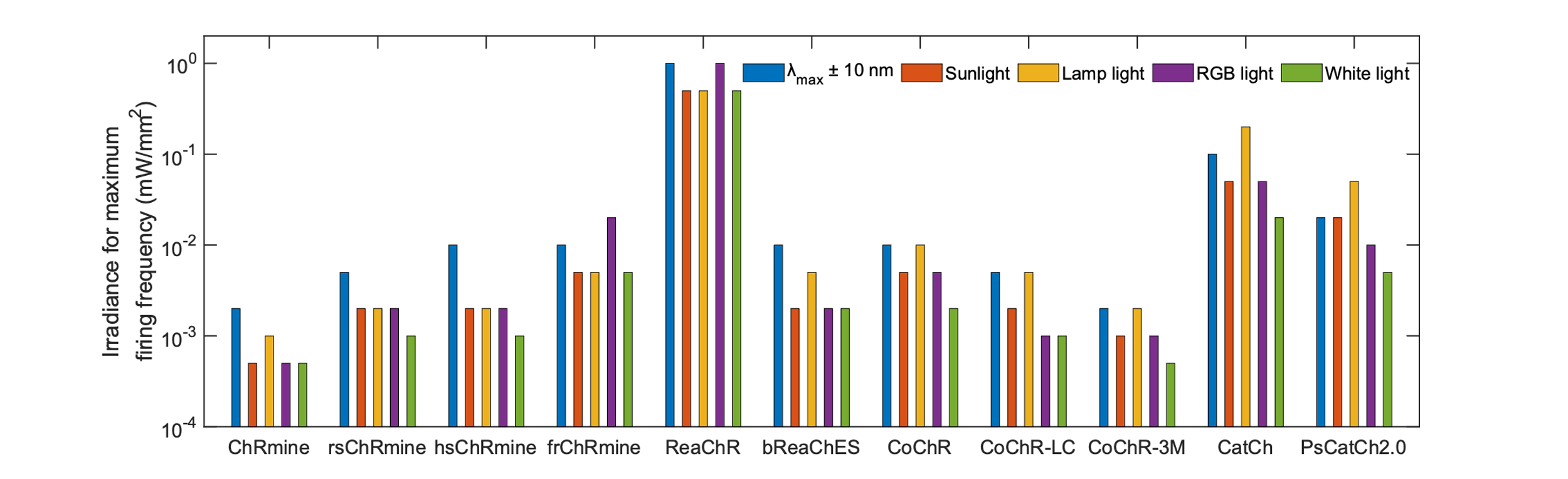


**Supplementary Fig. S5 Theoretical simulation of irradiance-dependence of firing response in different opsin-expressing RGNs on optogenetic excitation with near monochromatic and broadband light sources for 1s light pulse**. Required irradiance to achieve maximum firing rate under continuous stimulation in different opsins.


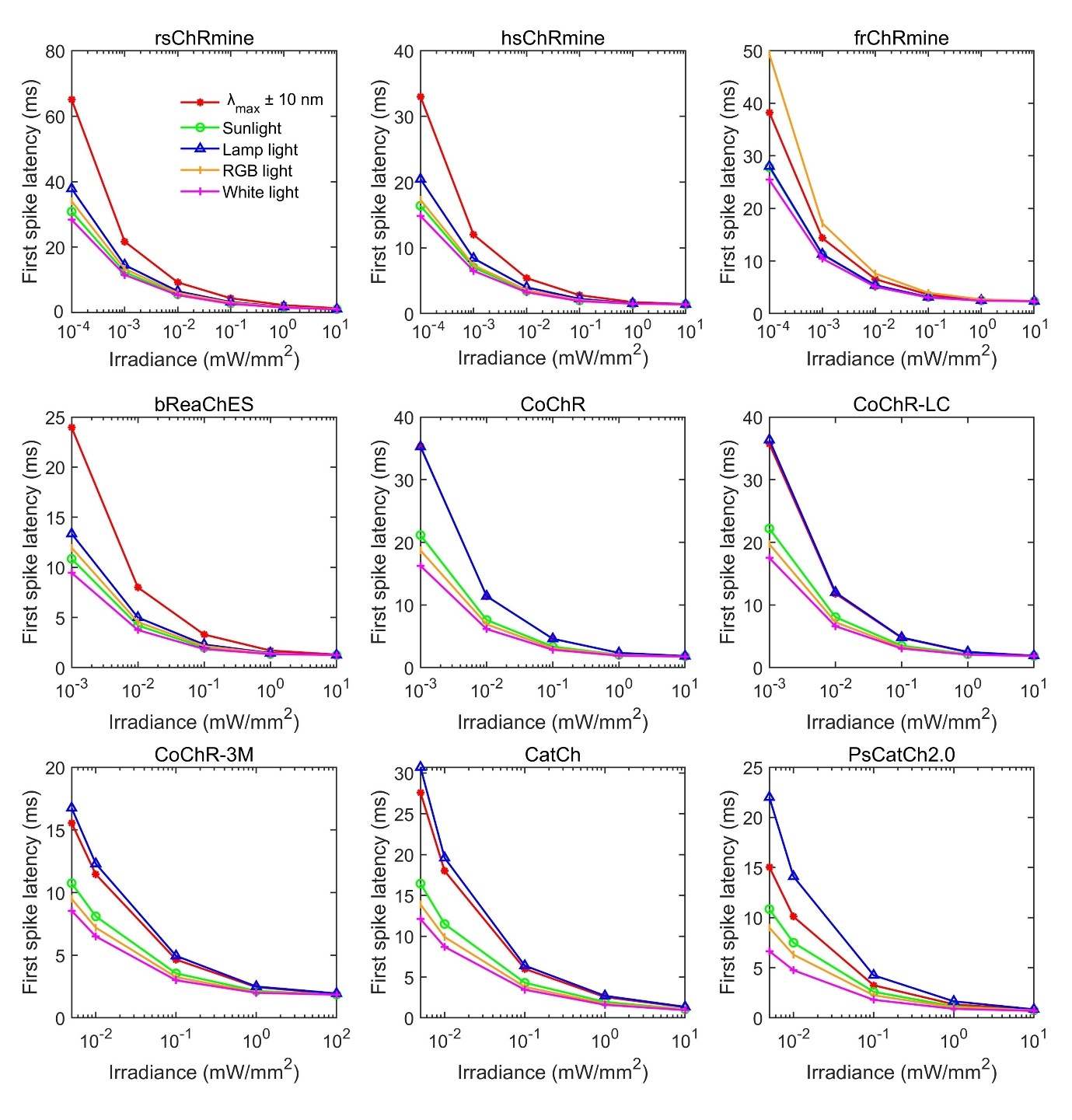


**Supplementary Fig. S6 Theoretical simulation of irradiance-dependence of the first spike latency in different opsin-expressing RGNs on optogenetic excitation with near monochromatic and broadband light sources for 1s light pulse**. Variation of the first spike latency with irradiance in different opsin-expressing neurons on illuminating 1s light pulse from different light sources.
